# Development and validation of ferroptosis-related lncRNAs prognosis signatures in kidney renal clear cell carcinoma

**DOI:** 10.1186/s12935-021-02284-1

**Published:** 2021-11-04

**Authors:** Xiao-Liang Xing, Zhi-Yong Yao, Jialan Ou, Chaoqun Xing, Feng Li

**Affiliations:** 1 The First Affiliated Hospital, Hunan University of Medicine, Huaihua, 418000 Hunan People’s Republic of China; 2 School of Public Health and Laboratory Medicine, Hunan University of Medicine, Huaihua, 418000 Hunan People’s Republic of China

**Keywords:** KIRC, Ferroptosis, lncRNA, Prognosis signatures

## Abstract

**Background:**

Ferroptosis is a recently recognised new type of cell death which may be a potential target for cancer therapy. In the present study, we aimed to screen ferroptosis-related differentially expressed long non-coding RNAs as biomarkers to predict the outcome of kidney renal clear cell carcinoma.

**Methods:**

RNAseq count data and corresponding clinical information were obtained from the Cancer Genome Atlas database. Lists of ferroptosis-related genes and long non-coding RNAs were obtained from the FerrDb and GENCODE databases, respectively. The candidate prognostic signatures were screened by Cox regression analyses and least absolute shrinkage and selection operator analyses.

**Results:**

Three ferroptosis-related long non-coding RNAs (DUXAP8, LINC02609, and LUCAT1) were significantly correlated with the overall survival of kidney renal clear cell carcinoma independently. Kidney renal clear cell carcinoma patients with high-risk values displayed worse OS. Meanwhile, the expression of these three ferroptosis-related long non-coding RNAs and their risk scores were significantly correlated with clinicopathological features. Principal component analyses showed that patients with kidney renal clear cell carcinoma have differential risk values were well distinguished by the three ferroptosis-related long non-coding RNAs.

**Conclusions:**

The present study suggests that the risk assessment model constructed by these three ferroptosis-related long non-coding RNAs could accurately predict the outcome of kidney renal clear cell carcinoma. We also provide a novel perspective for cancer prognosis screening.

**Supplementary Information:**

The online version contains supplementary material available at 10.1186/s12935-021-02284-1.

## Introduction

Kidney cancer is a cancer that originates in a kidney. It is also the most commonly diagnosed cancer in both men and women. In recent years, the incidence of kidney cancer has increased, mainly due to the aging process and tobacco use [1, 2]. Kidney renal clear cell carcinoma (KIRC) is the most common type of kidney cancer, accounting for approximately 75% of all kidney cancer diagnoses [3]. Approximately 40% of patients with advanced cancers eventually develop metastases, despite receiving surgical treatments [4]. Therefore, it is necessary to conduct more research on relevant prognostic signatures and possible therapeutic targets.

Ferroptosis was first proposed by Dixon in 2012 as type of novel cell death. It is defined as an iron-dependent and reactive oxygen species (ROS)-dependent cell death [5]. It is mainly characterised by cytological changes, including decreased or diminished mitochondrial cristae, ruptured outer mitochondrial membrane, or condensed mitochondrial membrane [6–10]. Previous studies have indicated that ferroptosis can be activated by diverse physiological conditions and pathological stresses [11]. Dysregulated ferroptosis is involved in multiple physiological and pathological processes, including cancer cell death [10]. Cumulative studies have demonstrated that dysregulated ferroptosis participates in several cancers, such as colorectal cancer, gastric cancer, and renal cancer [12–14]. Deregulated ferroptosis is increasingly recognised as an adaptive feature with the potential to eliminate malignant cells; moreover, it plays a pivotal role in inhibiting tumorigenesis [15]. Researchers have begun the process of employing the regulation of ferroptosis in tumour cells as a novel therapeutic approach [16–18]. Chemotherapy agents combined with ferroptosis inducers, such as erastin, have a significant synergistic antitumour effect. Notably, patients’ prognoses are with this approach better than those undergoing conventional chemotherapy alone [7].

Relatedly, long non-coding RNAs (lncRNAs) are a type of non-coding RNAs that are more than 200 nucleotides in length and account for nearly 70% of the human transcriptome [19]. This is important as lncRNAs play an integral role in several physiological and pathological cellular processes [20, 21]. Similar to dysregulated ferroptosis, dysregulated lncRNAs are closely related to cell proliferation, apoptosis, migration, and invasion in several different cancers [22–24]. Cumulative studies have demonstrated that lncRNAs are related to the overall survival of cancers. This means that they could be used as prognostic signatures to predict outcomes [25, 26]. Additionally, lncRNAs are increasingly recognised as crucial mediators in the regulation of ferroptosis [27].

It is important to recall the close relationship that ferroptosis has with the treatment and prognosis of cancers. Based on that and the fact that lncRNAs are key mediators in regulating ferroptosis, we speculated that several ferroptosis-related differentially expressed lncRNAs (FR-DELs) may be used as prognostic signatures for KIRC. Therefore, the aim of the present study was to identify suitable FR-DELs that can predict the prognostic outcome for patients living with KIRC. Through a series of bioinformatics analyses, we identified three FR-DELs (DUXAP8, LINC02609, and LUCAT1) that could be used as prognostic signatures for KIRC. We also verified the prediction of these three FR-DELs and the risk assessment model in the validation dataset as well as in the entire dataset.

## Methods

### Data processing

RNA-seq counts data of 72 controls and 530 patients with KIRC and their corresponding KIRC data were obtained from an open database, The Cancer Genome Atlas (TCGA) (https://portal.gdc.cancer.gov/). Annotation of lncRNAs was obtained from GENCODE (https://www.gencodegenes.org/). A list of ferroptosis-related genes (259) was obtained from FerrDb (http://www.zhounan.org/ferrdb).

We used the DESeq2 package in R 3.6.2 to screen the differentially expressed genes (DEGs) with the specific criterion baseMean  ≥ 100, ∣Log_2_FoldChange∣  ≥ 1.0, adj.p  < 0.05. Spearman correlation analyses were used to investigate the correlation of FR-DEGs and DELs with the specific criterion ∣r∣  ≥ 0.5, p  < 0.05.

### Sample processing

To identify suitable signatures and verify them, KIRC samples were randomly divided into training and validation groups (Table 1). We placed the patients without clear clinicopathological features into the unknown group. We excluded patients with unknown clinicopathological features from the overall samples. Considering the factors of sample size and analysis methods (discontinuous variables), we combined components with similar clinical phenotypes. For example, we regrouped the KIRC patients into two different groups based on the clinicopathologic features, such as T1  +  2 group and T3  +  4 group, N0 group and N1 group, M0 group and M1 group, Stage 1  +  2 group, and Stage 3  +  4 group in the overall survival analyses.

**Table 1:** Characteristics of KIRC patients

Characteristics	Entire group (n = 530)	Training group (n = 265)	Validation group (n = 265)
Age, years			
≤ 65	348	174	174
> 65	182	91	91
Gender			
Female	186	92	94
Male	344	173	171
Stage			
Stage I	265	141	124
Stage II	57	30	27
Stage III	123	58	65
Stage IV	82	35	47
Unknown	3	1	2
T			
T1	271	144	127
T2	69	35	34
T3	179	82	97
T4	11	4	7
N			
N0	239	122	117
N1	16	6	10
Unknown	275	137	138
M			
M0	422	217	205
M1	78	33	45
Unknown	30	15	15
Vital			
Alive	357	187	170
Death	173	78	95

### Development of lncRNAs as prognosis signatures

After regrouping by the median value, we evaluated each FR-DEL using univariate Cox regression analyses and Kaplan–Meier (K–M) analyses. We performed least absolute shrinkage and selection operator (LASSO) regression to avoid overfitting. We performed multivariate Cox regression analyses to identify suitable FR-DELs as prognostic signatures.

### Risk assessment model construction

After the candidate prognostic signatures were filtered by the analyses of univariate Cox regression, K–M, LASSO regression, and multivariate Cox regression, we constructed a risk assessment model using the following formula: Risk Value  =  β_FR-DEL1_  ×  Expression_FR-DEL1_  +  β_FR-DEL2_  ×  Expression_FR-DEL2_…  +  β_FR-DELn_  ×  Expression_FR-DELn_. After regrouping by the optimal cutoff value, univariate and multivariate Cox regression analyses were used to assess the prognostic value of the risk value model.

### Principal component analyses and functional enrichment

Principal component analyses (PCA) were conducted to reduce the dimensions of the study. PCA allowed us to visualise KIRC patients who had different risk values as defined by different DEGs. We found these by filtering using differentially expressed analyses, including 62 FR-DEGs filtered by differentially expressed analyses, 361 DELs filtered by differentially expressed analyses, 46 FR-DEGs filtered by Spearman analyses, 251 FR-DELs filtered by Spearman analyses, 9 FR-DELs filtered by univariate Cox, K–M, and LASSO analyses, and four FR-DELs after filtering by multivariate Cox analyses.

David 6.8 was used to carry out Gene Ontology (GO) and Kyoto Encyclopedia of Genes and Genomes (KEGG) analyses (https://david.ncifcrf.gov/).

### Statistical analyses

A repeated measure ANOVA followed by an unpaired two-tailed student’s t test was used as indicated. All results are expressed as the mean  ±  SEM.

## Results

### Differential expression analyses

**Fig. 1:**
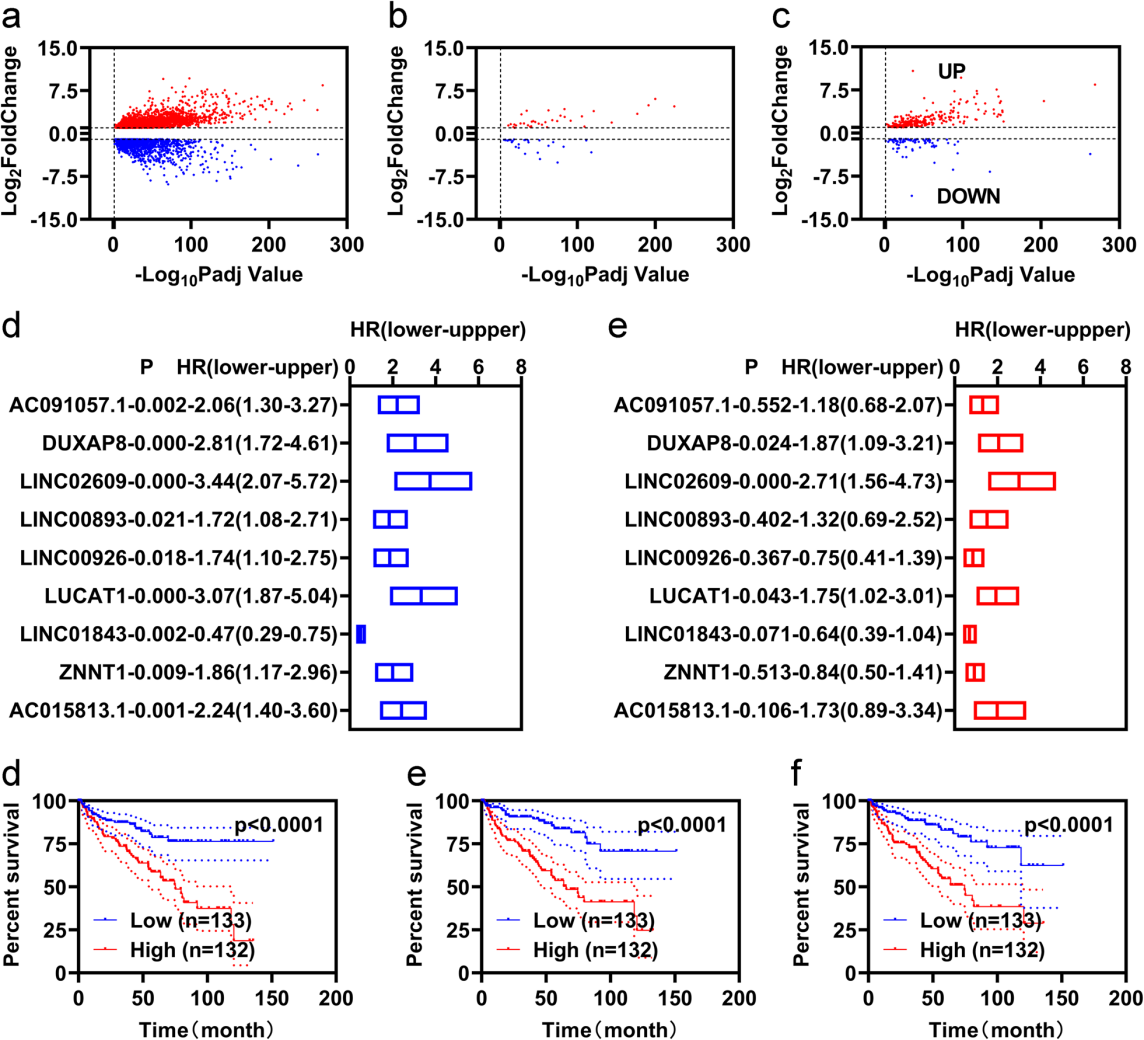
Differential expression analyses. Differential expression analyses of KIRC (**a** DEGs. **b** FR-DEG. **c** DELs). **d** Univariate Cox regression and K–M analyses illustrated nine FR-DELs associated with prognosis. **g** Multivariate Cox regression independently illustrated three FR-DELs associated with prognosis. **d**–**f** K–M plots of those 3 FR-DELs [DUXAP8 (**d**), LINC02609 (**e**), and LUCAT1(**f**)]

Through differential expression analyses, we screened 3978 DEGs, including 2573 upregulated DEGs and 1405 downregulated DEGs (Fig. 1a). By overlapping the 3978 DEGs with the ferroptosis genes and lncRNAs, we obtained 62 FR-DEGs (36 upregulated and 26 downregulated) and 361 DELs (278 upregulated and 83 downregulated), respectively (Fig. 1b, c). To obtain FR-DELs, we performed Spearman correlation analyses for the 62 FR-DEGs and 361 DELs. From this, we obtained 672 pairs of DELs-FR-DEGs which included 251 DELs and 46 FR-DEGs (Additional file 1: Table S1). We named these 251 DELs qw 251 FR-DELs.

### Development and validation of prognosis lncRNAs signatures

We first regrouped all KIRC patients into training and validation groups randomly. Clinical characteristics are displayed in Table 1. For preliminary screening, we found that 89 FR-DELs were correlated with the overall survival (OS) of patients with KIRC by univariate Cox analyses and K–M analysis in the training group (Additional file 1: Table S2). To avoid overfitting, we introduced LASSO analyses for the 89 FR-DELs (Additional file 1: Figure S1a, b) and obtained nine FR-DELs (Fig. 1d). Subsequently, we performed multivariate Cox analyses for the nine FR-DELs and identified three FR-DELs (DUXAP8, LINC02609, and LUCAT1) were independently correlated with the OS of patients with KIRC (Fig. 1e). Patients with KIRC with high expression of DUXAP8, LINC02609, and LUCAT1 displayed worse OS (Fig. 1d–f).

**Fig. 2:**
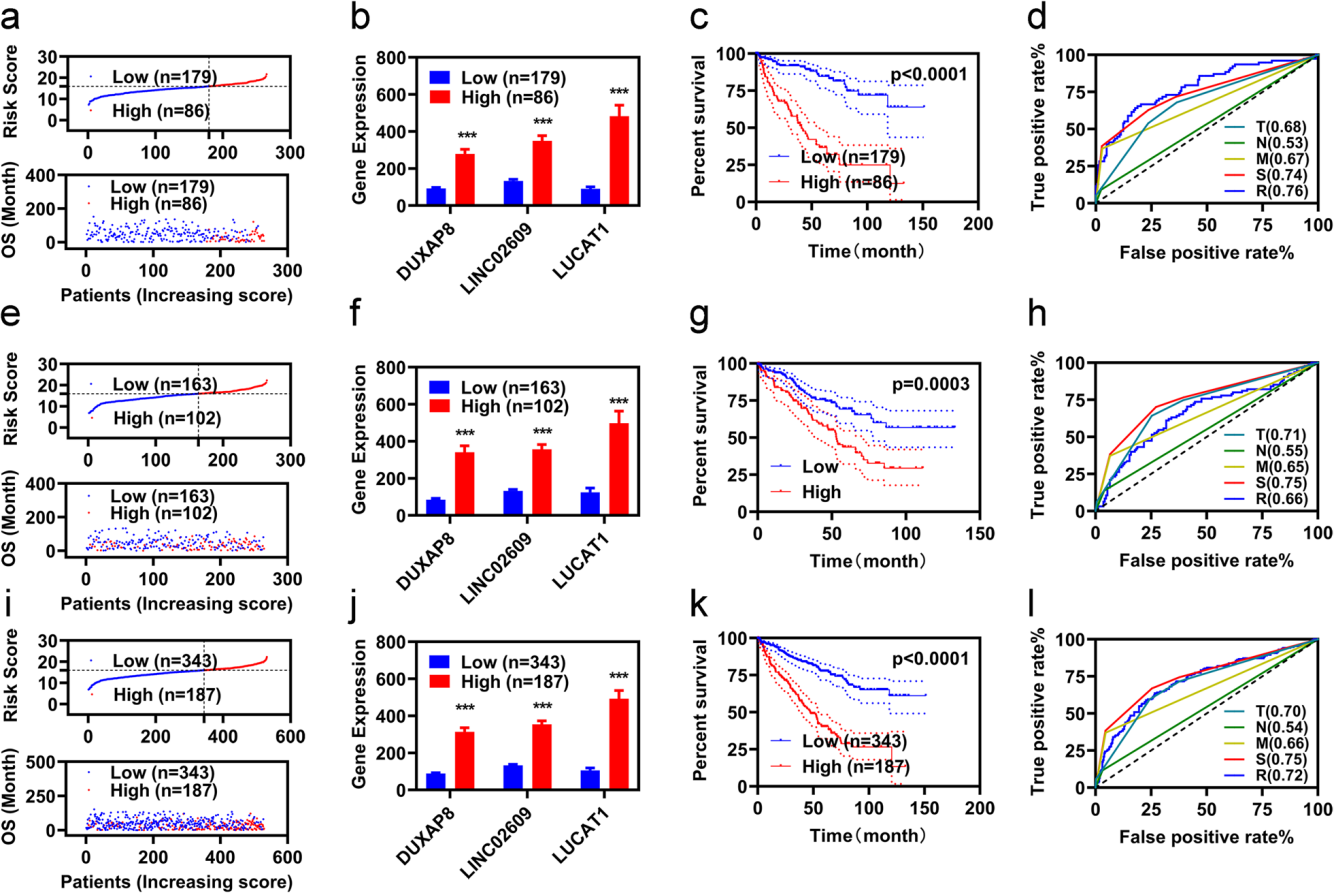
Development and validation of prognosis lncRNAs signature. Risk value and survival status (**a**), expression (**b**), K–M curve (**c**), ROC curve (**d**) of the prognostic signature in the training group. Risk value and survival status (**e**), expression (**f**), K–M curve (**g**), and ROC curve (**h**) of the prognostic signature in the validation group. Risk value and survival status (**i**), expression (**j**), K–M curve (**k**), and ROC curve (**l**) of the prognostic signature in the entire group. *p  < 0.05, **p  < 0.01, ***p  < 0.001

After employing multivariate Cox analyses, we constructed a risk assessment model using the three FR-DELs (DUXAP8, LINC02609, and LUCAT1). We used the optimal cut off value to regroup the KIRC patients (Additional file 1: Figure S2). The risk value (up) and survival status (down) of each KIRC patient are shown in Fig. 2a. The expression levels of the three FR-DELs (DUXAP8, LINC02609, and LUCAT1) in the differential risk groups are shown in Fig. 2b. The K–M analyses showed that patients with KIRC with high-risk values displayed worse OS (Fig. 2c). We then performed receiver operating characteristic (ROC) curves to assess the accuracy of the risk assessment model. The area under the curve (AUC) of the risk assessment model was comparable with the pathologic TNM and pathologic stage (Fig. 2d).

Subsequently, we performed the same studies on patients with KIRC in the validation group and the entire group. Similar results were observed in the validation group as well as the entire group (Fig. 2e–l).

### Independent prognostic factors of OS

To determine the role of the risk assessment model and clinicopathological features in prognostic prediction, K–M and multivariate Cox regression analyses were performed. The variable clinicopathological features included age, sex, pathologic TNM, and pathologic stage.

**Fig. 3:**
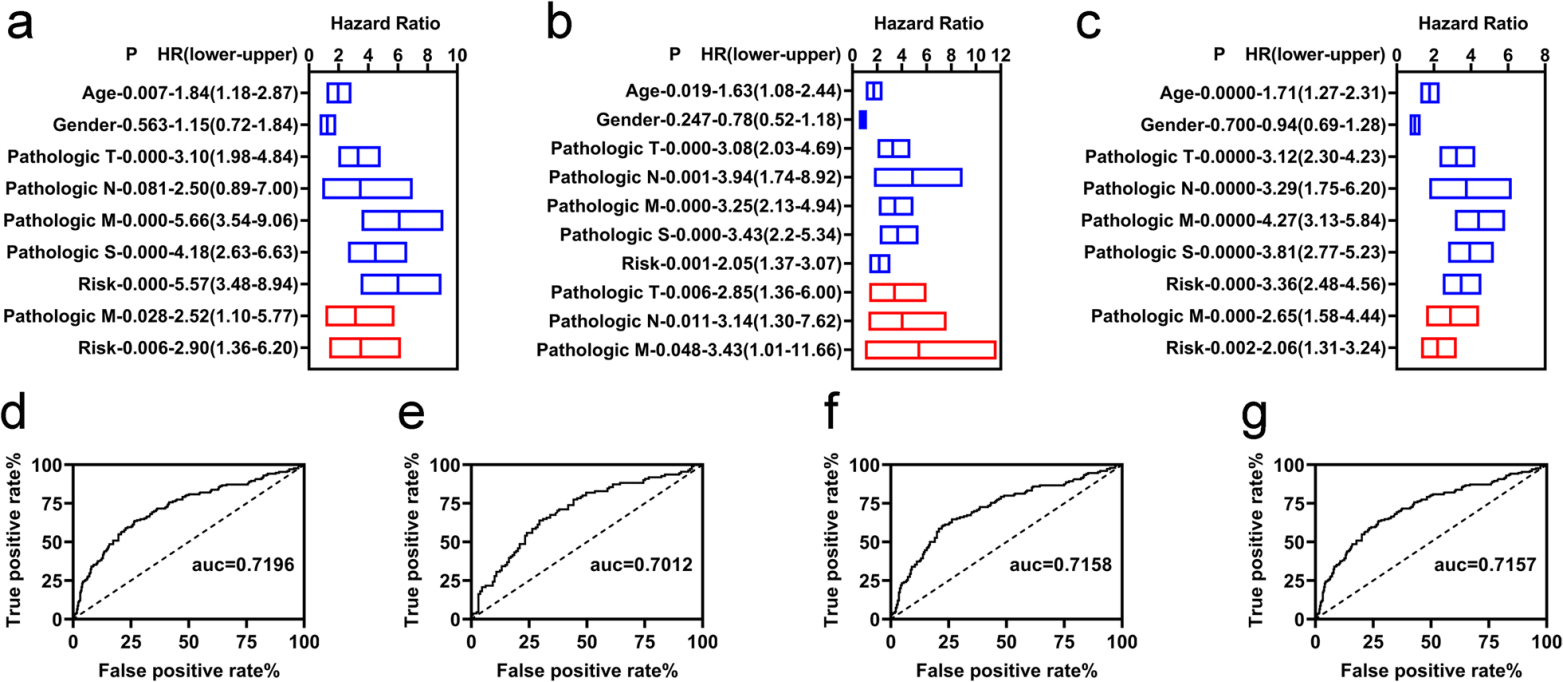
Independent prognostic factors of overall survival. **a**–**c** Univariate (blue) and multivariate (red) Cox regression of prognostic factors in the training, validation, and entire groups, respectively. ROC curve plot of risk model for all patients (**d**), patients at 3 (**e**), 5 (**f**), and 10-year (**g**) in the entire group

In the training group, we found that age, pathologic T, pathologic M, pathologic stage, and risk model were correlated with OS by K–M analyses (Fig. 3a). The pathologic M and the risk model were still correlated with OS by multivariate Cox analyses (Fig. 3a). In the validation and entire group, we also found that age, pathologic T, pathologic N, pathologic M, overall pathologic stage, and risk model were correlated with OS by K–M analyses (Fig. 3b, c). The pathologic TNM was still correlated with the OS in the validation group (Fig. 3b). Meanwhile, the pathologic M and the risk model were still correlated with OS by multivariate Cox analyses (Fig. 3c).

We performed ROC curve analyses to assess the accuracy of the risk assessment model for all patients and patients at 3, 5, and 10-year in the entire group. All AUC values were over 0.7 (Fig. 3d–f).

### Correlation analyses with clinicopathological features

**Fig. 4:**
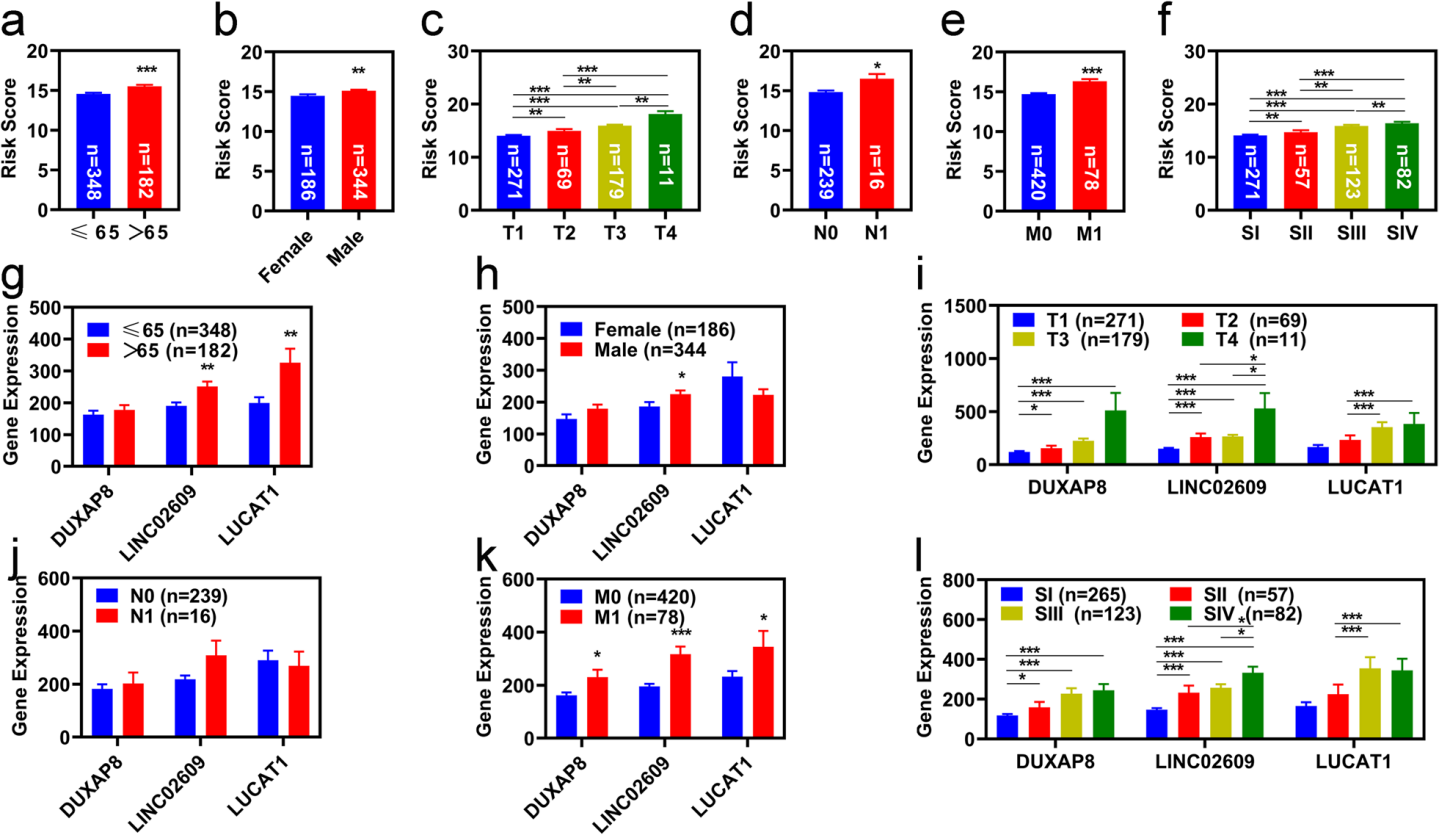
Correlation analyses with clinicopathological features. Correlation of risk value with clinicopathological features [age (**a)**, sex (**b**), pathologic T (**c**), pathologic N (**d**), pathologic M (**e**), and pathologic stage (**f**)]. Correlation of expression of ferroptosis-related lncRNAs with clinicopathological features [age (**a**), sex (**b**), pathologic T (**c**), pathologic N (**d**), pathologic M (**e**), and pathologic stage (**f**)]. *p  < 0.05, **p  < 0.01, ***p  < 0.001

We explored the relationship of the risk value and the clinicopathological features in the entire group. In doing so, we found that the risk values differed significantly among patients with different clinicopathological features. Risk factors increased significantly in KIRC patients over 65 years of age who were male, had pathologic N1, and fell under the pathologic M1 groups (Fig. 4a, b, d, e). There were also significant differences in KIRC patients with different pathologic T and pathologic stages (Fig. 4c, f).

We also explored the relationship between the expression of these three FR-DELs and their clinicopathological features. The results are displayed in Fig. 4g–l. These results indicated that the expression of these three FR-DELs was significantly different among the different clinicopathological features.

### Functional enrichment and PCA

**Fig. 5:**
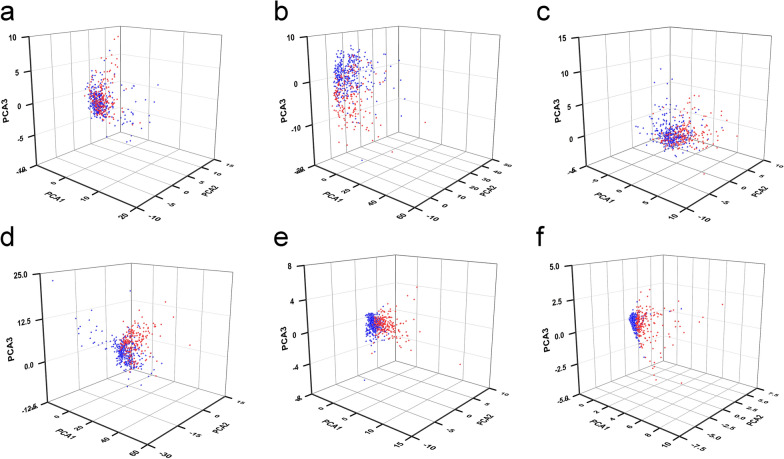
Principal component analyses. Principal component analysis plots displayed the distribution of patients with KIRC with high and low risk values based on 62 FR-DEGs (**a**), 361 DELs (**b**), 46 FR-DEGs (**c**), 251 FR-DELs (**d**), 9 FR-DELs (**e**), and 3 FR-DELs (**f**). Blue means low risk. Red means high risk

We performed PCA analyses to explore the distribution of each case with a differential risk value. PCA analyses were performed using the 62 FR-DEGs, 361 DELs, 46 FR-DEGs, 251 FR-DELs, and nine FR-DELs. The results indicated that the KIRC patients with low-risk values could be largely separated from the KIRC patients with high-risk values (Fig. 5a–e). In particular, we clearly distinguished the high-risk values from the low-risk values in patients with KIRC using the three FR-DELs as prognostic signatures (Fig. 5f).

**Fig. 6:**
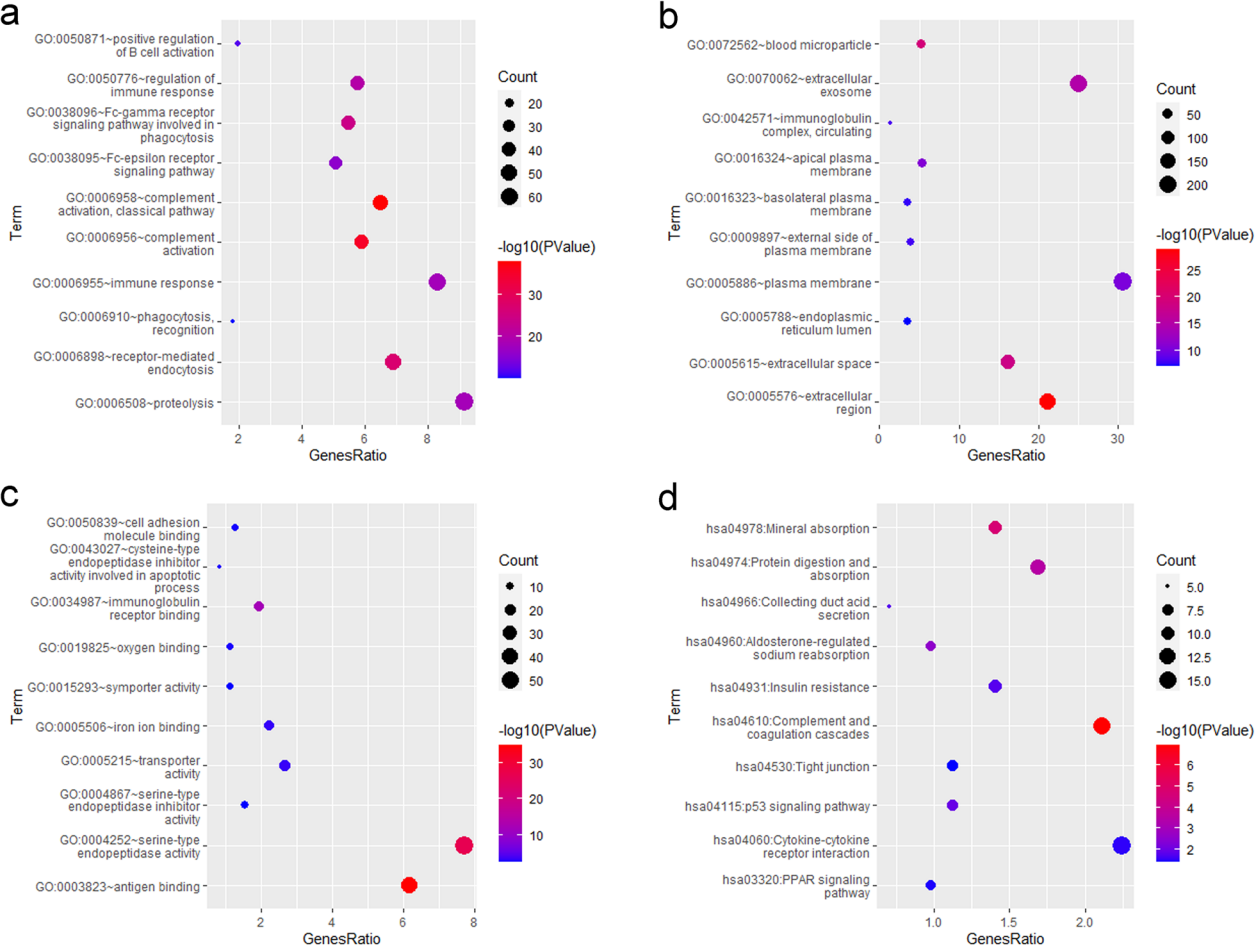
Functional enrichment analyses. Significantly enriched GO term (top 10). *BP* biological process (**a**) *CC* cellular component (**b**). *MF* molecular function (**c**). **d** Significantly enriched KEGG pathway (top 10)

To explore the gene expression status between the KIRC patients in the high-risk group and low-risk group, we performed differentially expressed analyses using DEseq2 and identified 757 DEGs, including 493 upregulated and 264 downregulated (Additional file 1: Figure S3). Then, we used David 6.8 to carry out GO and KEGG enrichment analyses (Fig. 6a–d). The KEGG analysis revealed that several signalling pathways related to cancer and ferroptosis were enriched, such as the p53 signalling pathway (Fig. 6d).

## Discussion

The main treatment for KIRC is surgery. However, almost 40% of patients with advanced KIRC who undergo surgery will eventually develop distant metastases [28, 29]. The overall survival of patients with metastatic KIRC is poor. Even prior to surgery, approximately 10% of KIRC patients survive for only 5 years [30]. Previous studies have suggested that even if the TNM stage or risk factors are the same, they will show different clinical outcomes due to molecular heterogeneity. Therefore, it is important to identify suitable prognostic molecular signatures [31]. Ferroptosis is a recognised novel form of programmed cell death which is involved in the migration, invasion, and proliferation of several cancers [32–35]. Additionally, several studies have demonstrated that lncRNAs play pivotal roles in the regulation of ferroptosis [36–40].

In the present study, we identified three FR-DELs (DUXAP8, LINC02609, and LUCAT1) by filtering using univariate Cox analyses, K–M analyses, LASSO regression analyses, and multivariate Cox analyses. We chose to use these as all of these could be used as prognostic signatures for KIRC. We constructed a risk assessment model using these three FR-DELs. KIRC patients with high-risk values displayed worse OS. ROC curve analyses also suggested that the AUC values of this model were over 0.7. Moreover, Cox analysis revealed that the risk model could be an independent prognostic factor. PCA analyses further revealed that KIRC patients with high-risk values were largely distinguishable from the KIRC patients with high-risk values.

Gong et al. found that DUXAP8 promotes proliferation, migration, and invasion via miR-490-5P, RAB14, HK2, LDHA, and EMT [41–44]. In renal cancer, Chen et al. found that overexpression of DUXAP8 promotes the growth of renal cancer [45, 46]. Huang et al. further found that overexpression of DUXAP8 promotes renal cell proliferation by downregulating miR-126 expression. In the present study, we found that DUXAP8 expression was significantly increased in patients with KIRC. These results reinforce the correlation between DUXAP8 and KIRC. In addition, we found that DUXAP8 was correlated with the OS of KIRC by LASSO regression, univariate Cox, K–M, and multivariate Cox analyses. KIRC patients with high DUAPX8 expression displayed worse OS. Chen et al. suggested that DUXAP8 may serve as a potential prognostic signature for renal cancer [45]. Our present results are consistent with previous studies which also suggested that DUXAP8 could be a suitable prognostic signature for KIRC.

He et al. found that LINC02609 was significantly associated with OS in 258 patients with sarcoma [47]. In the present study, we found that the expression of LINC02609 was significantly increased in KIRC patients while it was also significantly increased in KIRC patients with high-risk values. KIRC patients with high expression of LINC02609 exhibited worse OS. LINC02609 may be a prognostic signature for KIRC. Additionally, Su et al. found that the expression of LINC02609 was increased not only in advanced stages and grades than in the early stages and grades, but also in the tissues of tumour and distant metastasis than in the normal and non-distant metastasis control [48]. That same study also found that LINC02609 has significant distant metastasis and prognostic potential [48]. In the present study, we found that the expression of LINC02609 was correlated with pathologic T, pathologic M, and pathologic stage. Our results were consistent with those of previous studies, reinforcing the correlation of LINC02609 with distant metastasis. Su et al. found that LINC02609 could be used as a prognostic signature for KIRC based on distant metastasis-related lncRNAs. In the present study, we found that LINC02609 could also be used as a prognostic signature for KIRC based on ferroptosis and Cox regression analyses. In the present study, we identified that LINC02609 could be a prognostic signature for KIRC, which further indicated the close correlation between LINC02609 and KIRC that has been alluded to in the existing literature.

Previous studies demonstrated that aberrant expression of LUCAT1 is correlated with several cancers. These include pancreatic cancer, ovarian cancer, bladder cancer, lung cancer, breast cancer, and renal cancer. Cao et al. found that LUCAT1 expression is increased in human pancreatic cancer cell lines. The high expression of LUCAT1 enhances the pathogenesis of pancreatic cancer and promotes the proliferation and invasion of pancreatic cancer cells by inducing the phosphorylation of Akt and p38 MAPK [49]. Liu et al. also found that the expression of LUCAT1 was highly expressed in ovarian cancer cell lines [50]. The proliferation rate of the LUCAT1 knockdown group was significantly decreased, while that of the LUCAT1 silencing group was significantly increased [50]. Chen et al. found that the downregulation of LUCAT1 could suppress the migration and invasion of bladder cancer by targeting miR-181c-5p [51]. Regarding renal cancer, Zheng et al. found that LUCAT1 expression was also significantly increased compared with normal tissue [52]. LUCAT1 promotes proliferation and invasion of renal cancer through the AKT/GSK-3β signalling pathway [52]. All of these studies indicate that LUCAT1 is closely correlated with the development of various cancers. Previous studies also demonstrated that LUCAT1 is correlated with OS and could be used as a prognostic signature for several cancers, such as papillary thyroid cancer, non-small lung cancer, and renal cancer. In the present study, we found that the expression of LUCAT1 was significantly increased in KIRC patients and significantly increased in KIRC patients with high-risk values. The results of our present study were consistent with those of previous studies, which reinforce the feasibility of LUCAT1 as a prognostic signature for KIRC [53].

Our present study identified three FR-DELs (DUXAP8, LINC02609, and LUCAT1) as candidates for prognostic signatures of KIRC. However, other studies have also identified a number of other genes that may serve as prognostic signatures for KIRC [54–57].

There are some limitations to the current study. For example, in this study, the control group was smaller than the experimental group. An increase in the sample size would enhance the credibility of these results. Another shortcoming of the current study is the lack of corroboration from clinical studies. This would be the focus of the authors’ future research. Through sample collection, candidate biomarker detection, and follow-up studies, we shall further clarify their feasibility as a prognostic biomarker of KIRC.

## Conclusions

Through a series of bioinformatics analyses, we identified three FR-DELs (DUXAP8, LINC02609, and LUCAT1) which can be used as prognostic signatures to predict the outcome of patients with KIRC. Our study presented a new research strategy for exploring the mechanism of ferroptosis. Moreover, this study provided an individualised prognostic prediction for patients with KIRC. However, there were some limitations to this study, mostly due to the lack of clinical validation.

## Supplementary Information

**Additional file 1: Table S1. **The correlation of DELs and FR-DEGs by Spearman analysis. **Table S2.** Verified overall survival correlated FR-DELs by univariate Cox analysis. **Figure S1.** LASSO regression analysis for those 89 FR-DELs verified by univariate Cox analyses and Kaplan-Meier analysis. **Figure S2.** The verified optimal cutoff value was 15.962. **Figure S3.** Volcano plot of DEGs for KIRC between high risk group and low risk group.

## Data Availability

The data that support the findings of this study are openly available in TCGA at https://portal.gdc.cancer.gov/.

## Abbreviations

FR: Ferroptosis-related
DELs: Differentially expressed log
DEGs: Differentially expressed genes
KIRC: Kidney renal clear cell carcinoma
FR-DELs: Ferroptosis-related differently expressed long
ROS: Reactive oxygen species
lncRNAs: Long non-coding RNAs
OS: Overall survival
TCGA: The Cancer Genome Atlas
LASSO: Least absolute shrinkage and selection operator
PCA: Principal component analyses
FR-DEGs: Ferroptosis-related differentially expressed genes
GO: Gene ontology
KEGG: Kyoto Encyclopedia of Genes and Genomes
ROC: Receiver operating characteristic
AUC: Area under curve
