# Supplementary material for: Development and validation of ferroptosis-related lncRNAs prognosis signatures in kidney renal clear cell carcinoma
Source: Cancer Cell Int. 2021 Nov 4;21:591. doi: 10.1186/s12935-021-02284-1 (PMC8567554; doi:10.1186/s12935-021-02284-1)
Supplement: Supplementary file 1 — Additional file 1: Table S1. The correlation of DELs and FR-DEGs by Spearman analysis. Table S2. Verified overall survival correlated FR-DELs by univariate Cox analysis. Figure S1. LASSO regression analysis for those 89 FR-DELs verified by univariate Cox analyses and Kaplan-Meier analysis. Figure S2. The verified optimal cutoff value was 15.962. Figure S3. Volcano plot of DEGs for KIRC between high risk group and low risk group. [file 12935_2021_2284_MOESM1_ESM.docx]

**Development and validation of ferroptosis-related lncRNAs prognosis signatures in kidney renal clear cell carcinoma**

Xiao-Liang Xing^1,2#*^, Zhi-Yong Yao^2#^, Jialan Ou^1^, Chaoqun Xing^1^, Feng Li^1,2*^

^1^The first Affiliated Hospital, Hunan University of Medicine, Huaihua 418000, Hunan, P. R. China.

^2^School of Public Health and Laboratory Medicine, Hunan University of Medicine, Huaihua 418000, Hunan, P. R. China.

#contributed equally to this work.

*corresponding Author: Feng Li, 646301252@qq.com; Xiao-Liang Xing, zhangjing@sklmg.edu.cn.

**Supplementary information:** 2 tables and 3 figures.

**Table 1 The correlation of DELs and FR-DEGs by Spearman analysis**

| **DEL** | **Fe-DEGs** | **P** | **R** | **DEL** | **Fe-DEGs** | **P** | **R** |
| --- | --- | --- | --- | --- | --- | --- | --- |
| ENSG00000082929 | ENSG00000176171 | 0.000 | 0.57 | ENSG00000247095 | ENSG00000102125 | 0.000 | 0.66 |
| ENSG00000082929 | ENSG00000107159 | 0.000 | 0.56 | ENSG00000247095 | ENSG00000108839 | 0.000 | 0.64 |
| ENSG00000082929 | ENSG00000135245 | 0.000 | 0.50 | ENSG00000247095 | ENSG00000135245 | 0.000 | 0.52 |
| ENSG00000172965 | ENSG00000015475 | 0.000 | 0.60 | ENSG00000247271 | ENSG00000089220 | 0.000 | 0.61 |
| ENSG00000172965 | ENSG00000105974 | 0.000 | 0.58 | ENSG00000247271 | ENSG00000120053 | 0.000 | 0.54 |
| ENSG00000172965 | ENSG00000166741 | 0.000 | 0.56 | ENSG00000247373 | ENSG00000102125 | 0.000 | 0.74 |
| ENSG00000172965 | ENSG00000059804 | 0.000 | 0.56 | ENSG00000247373 | ENSG00000108839 | 0.000 | 0.65 |
| ENSG00000172965 | ENSG00000115902 | 0.000 | 0.55 | ENSG00000247400 | ENSG00000233237 | 0.000 | 0.62 |
| ENSG00000172965 | ENSG00000140464 | 0.000 | 0.55 | ENSG00000247774 | ENSG00000012779 | 0.000 | 0.64 |
| ENSG00000172965 | ENSG00000168209 | 0.000 | 0.51 | ENSG00000247774 | ENSG00000116701 | 0.000 | 0.63 |
| ENSG00000172965 | ENSG00000101255 | 0.000 | 0.51 | ENSG00000247774 | ENSG00000140464 | 0.000 | 0.61 |
| ENSG00000172965 | ENSG00000147889 | 0.000 | 0.50 | ENSG00000247774 | ENSG00000015475 | 0.000 | 0.59 |
| ENSG00000174171 | ENSG00000108839 | 0.000 | 0.51 | ENSG00000247774 | ENSG00000165168 | 0.000 | 0.57 |
| ENSG00000174171 | ENSG00000102125 | 0.000 | 0.50 | ENSG00000247774 | ENSG00000171848 | 0.000 | 0.54 |
| ENSG00000177133 | ENSG00000155066 | 0.000 | 0.57 | ENSG00000247774 | ENSG00000026508 | 0.000 | 0.54 |
| ENSG00000177410 | ENSG00000101255 | 0.000 | 0.62 | ENSG00000247774 | ENSG00000179593 | 0.000 | 0.53 |
| ENSG00000177410 | ENSG00000107159 | 0.000 | 0.56 | ENSG00000247774 | ENSG00000147889 | 0.000 | 0.52 |
| ENSG00000177410 | ENSG00000015475 | 0.000 | 0.55 | ENSG00000247774 | ENSG00000118503 | 0.000 | 0.52 |
| ENSG00000177410 | ENSG00000105974 | 0.000 | 0.53 | ENSG00000247982 | ENSG00000102125 | 0.000 | 0.73 |
| ENSG00000177410 | ENSG00000117394 | 0.000 | 0.53 | ENSG00000247982 | ENSG00000108839 | 0.000 | 0.59 |
| ENSG00000179406 | ENSG00000102125 | 0.000 | 0.75 | ENSG00000248019 | ENSG00000102125 | 0.000 | 0.70 |
| ENSG00000179406 | ENSG00000108839 | 0.000 | 0.69 | ENSG00000248019 | ENSG00000108839 | 0.000 | 0.69 |
| ENSG00000180769 | ENSG00000089220 | 0.000 | 0.59 | ENSG00000248019 | ENSG00000112715 | 0.000 | 0.60 |
| ENSG00000180769 | ENSG00000120053 | 0.000 | 0.59 | ENSG00000248323 | ENSG00000059804 | 0.000 | 0.62 |
| ENSG00000180769 | ENSG00000100253 | 0.000 | 0.52 | ENSG00000248323 | ENSG00000101255 | 0.000 | 0.62 |
| ENSG00000181577 | ENSG00000112715 | 0.000 | 0.67 | ENSG00000248323 | ENSG00000166741 | 0.000 | 0.54 |
| ENSG00000183784 | ENSG00000100253 | 0.000 | 0.66 | ENSG00000248323 | ENSG00000099194 | 0.000 | 0.53 |
| ENSG00000183784 | ENSG00000100889 | 0.000 | 0.53 | ENSG00000248323 | ENSG00000117394 | 0.000 | 0.52 |
| ENSG00000186056 | ENSG00000102125 | 0.000 | 0.69 | ENSG00000248323 | ENSG00000171848 | 0.000 | 0.50 |
| ENSG00000186056 | ENSG00000108839 | 0.000 | 0.58 | ENSG00000248636 | ENSG00000153714 | 0.000 | 0.51 |
| ENSG00000186056 | ENSG00000112715 | 0.000 | 0.55 | ENSG00000248866 | ENSG00000198793 | 0.000 | 0.53 |
| ENSG00000187621 | ENSG00000100253 | 0.000 | 0.51 | ENSG00000248866 | ENSG00000233237 | 0.000 | 0.52 |
| ENSG00000187951 | ENSG00000171848 | 0.000 | 0.59 | ENSG00000249087 | ENSG00000102125 | 0.000 | 0.65 |
| ENSG00000187951 | ENSG00000102125 | 0.000 | 0.52 | ENSG00000249087 | ENSG00000108839 | 0.000 | 0.54 |
| ENSG00000187951 | ENSG00000108839 | 0.000 | 0.51 | ENSG00000249550 | ENSG00000101255 | 0.000 | 0.54 |
| ENSG00000188185 | ENSG00000102125 | 0.000 | 0.58 | ENSG00000249550 | ENSG00000171848 | 0.000 | 0.53 |
| ENSG00000188185 | ENSG00000108839 | 0.000 | 0.51 | ENSG00000249669 | ENSG00000112715 | 0.000 | 0.57 |
| ENSG00000188511 | ENSG00000115902 | 0.000 | 0.56 | ENSG00000249776 | ENSG00000147872 | 0.000 | 0.51 |
| ENSG00000196167 | ENSG00000233237 | 0.000 | 0.60 | ENSG00000249776 | ENSG00000176171 | 0.000 | 0.50 |
| ENSG00000196295 | ENSG00000102125 | 0.000 | 0.73 | ENSG00000249859 | ENSG00000102125 | 0.000 | 0.65 |
| ENSG00000196295 | ENSG00000108839 | 0.000 | 0.67 | ENSG00000249859 | ENSG00000101255 | 0.000 | 0.58 |
| ENSG00000196295 | ENSG00000112715 | 0.000 | 0.51 | ENSG00000249859 | ENSG00000015475 | 0.000 | 0.57 |
| ENSG00000196668 | ENSG00000108839 | 0.000 | 0.69 | ENSG00000249859 | ENSG00000107159 | 0.000 | 0.56 |
| ENSG00000196668 | ENSG00000112715 | 0.000 | 0.64 | ENSG00000249859 | ENSG00000147889 | 0.000 | 0.53 |
| ENSG00000196668 | ENSG00000102125 | 0.000 | 0.61 | ENSG00000249859 | ENSG00000166741 | 0.000 | 0.51 |
| ENSG00000196696 | ENSG00000102125 | 0.000 | 0.71 | ENSG00000249859 | ENSG00000140464 | 0.000 | 0.50 |
| ENSG00000196696 | ENSG00000108839 | 0.000 | 0.67 | ENSG00000250303 | ENSG00000147872 | 0.000 | 0.54 |
| ENSG00000196756 | ENSG00000102125 | 0.000 | 0.67 | ENSG00000251136 | ENSG00000102125 | 0.000 | 0.63 |
| ENSG00000197182 | ENSG00000102125 | 0.000 | 0.59 | ENSG00000251169 | ENSG00000147872 | 0.000 | 0.62 |
| ENSG00000197536 | ENSG00000102125 | 0.000 | 0.62 | ENSG00000251169 | ENSG00000176171 | 0.000 | 0.57 |
| ENSG00000197536 | ENSG00000108839 | 0.000 | 0.57 | ENSG00000251320 | ENSG00000105974 | 0.000 | 0.58 |
| ENSG00000197536 | ENSG00000112715 | 0.000 | 0.51 | ENSG00000251320 | ENSG00000168209 | 0.000 | 0.51 |
| ENSG00000197989 | ENSG00000102125 | 0.000 | 0.70 | ENSG00000251320 | ENSG00000101255 | 0.000 | 0.51 |
| ENSG00000197989 | ENSG00000108839 | 0.000 | 0.58 | ENSG00000251320 | ENSG00000166741 | 0.000 | 0.50 |
| ENSG00000197989 | ENSG00000168209 | 0.000 | 0.52 | ENSG00000251432 | ENSG00000102125 | 0.000 | 0.62 |
| ENSG00000203392 | ENSG00000112715 | 0.000 | 0.67 | ENSG00000251442 | ENSG00000165168 | 0.000 | 0.79 |
| ENSG00000203392 | ENSG00000108839 | 0.000 | 0.65 | ENSG00000251442 | ENSG00000116701 | 0.000 | 0.75 |
| ENSG00000203392 | ENSG00000102125 | 0.000 | 0.63 | ENSG00000251442 | ENSG00000171848 | 0.000 | 0.57 |
| ENSG00000203497 | ENSG00000100292 | 0.000 | 0.50 | ENSG00000251442 | ENSG00000179593 | 0.000 | 0.57 |
| ENSG00000204261 | ENSG00000102125 | 0.000 | 0.56 | ENSG00000251442 | ENSG00000012779 | 0.000 | 0.57 |
| ENSG00000204362 | ENSG00000112715 | 0.000 | 0.59 | ENSG00000251442 | ENSG00000026508 | 0.000 | 0.55 |
| ENSG00000204362 | ENSG00000106327 | 0.000 | 0.58 | ENSG00000251442 | ENSG00000100292 | 0.000 | 0.55 |
| ENSG00000204362 | ENSG00000135245 | 0.000 | 0.56 | ENSG00000251562 | ENSG00000102125 | 0.000 | 0.58 |
| ENSG00000204362 | ENSG00000166741 | 0.000 | 0.52 | ENSG00000251562 | ENSG00000108839 | 0.000 | 0.57 |
| ENSG00000204362 | ENSG00000059804 | 0.000 | 0.52 | ENSG00000253574 | ENSG00000147872 | 0.000 | 0.54 |
| ENSG00000204362 | ENSG00000117394 | 0.000 | 0.51 | ENSG00000253574 | ENSG00000176171 | 0.000 | 0.52 |
| ENSG00000204362 | ENSG00000107159 | 0.000 | 0.50 | ENSG00000253764 | ENSG00000102125 | 0.000 | 0.57 |
| ENSG00000204362 | ENSG00000101255 | 0.000 | 0.50 | ENSG00000253764 | ENSG00000112715 | 0.000 | 0.54 |
| ENSG00000204528 | ENSG00000108839 | 0.000 | 0.58 | ENSG00000253764 | ENSG00000108839 | 0.000 | 0.51 |
| ENSG00000204528 | ENSG00000112715 | 0.000 | 0.58 | ENSG00000253837 | ENSG00000112715 | 0.000 | 0.53 |
| ENSG00000204677 | ENSG00000112715 | 0.000 | 0.52 | ENSG00000254154 | ENSG00000108839 | 0.000 | 0.68 |
| ENSG00000204677 | ENSG00000108839 | 0.000 | 0.50 | ENSG00000254154 | ENSG00000112715 | 0.000 | 0.58 |
| ENSG00000205885 | ENSG00000108839 | 0.000 | 0.70 | ENSG00000254528 | ENSG00000233237 | 0.000 | 0.55 |
| ENSG00000205885 | ENSG00000112715 | 0.000 | 0.59 | ENSG00000254528 | ENSG00000120053 | 0.000 | 0.51 |
| ENSG00000206195 | ENSG00000101255 | 0.000 | 0.51 | ENSG00000254815 | ENSG00000102125 | 0.000 | 0.74 |
| ENSG00000206195 | ENSG00000015475 | 0.000 | 0.51 | ENSG00000254815 | ENSG00000108839 | 0.000 | 0.73 |
| ENSG00000206344 | ENSG00000108839 | 0.000 | 0.76 | ENSG00000254815 | ENSG00000112715 | 0.000 | 0.59 |
| ENSG00000206344 | ENSG00000102125 | 0.000 | 0.68 | ENSG00000255026 | ENSG00000102125 | 0.000 | 0.55 |
| ENSG00000206344 | ENSG00000112715 | 0.000 | 0.64 | ENSG00000255026 | ENSG00000108839 | 0.000 | 0.54 |
| ENSG00000214145 | ENSG00000135245 | 0.000 | 0.61 | ENSG00000255462 | ENSG00000166741 | 0.000 | 0.57 |
| ENSG00000214145 | ENSG00000168209 | 0.000 | 0.56 | ENSG00000255462 | ENSG00000107159 | 0.000 | 0.53 |
| ENSG00000214145 | ENSG00000107159 | 0.000 | 0.56 | ENSG00000255462 | ENSG00000176171 | 0.000 | 0.51 |
| ENSG00000214145 | ENSG00000112715 | 0.000 | 0.55 | ENSG00000255717 | ENSG00000102125 | 0.000 | 0.59 |
| ENSG00000214145 | ENSG00000166741 | 0.000 | 0.51 | ENSG00000255717 | ENSG00000108839 | 0.000 | 0.52 |
| ENSG00000214783 | ENSG00000108839 | 0.000 | 0.68 | ENSG00000255717 | ENSG00000112715 | 0.000 | 0.52 |
| ENSG00000214783 | ENSG00000102125 | 0.000 | 0.67 | ENSG00000255737 | ENSG00000101255 | 0.000 | 0.60 |
| ENSG00000214783 | ENSG00000112715 | 0.000 | 0.58 | ENSG00000255737 | ENSG00000140464 | 0.000 | 0.55 |
| ENSG00000214922 | ENSG00000108839 | 0.000 | 0.68 | ENSG00000255737 | ENSG00000015475 | 0.000 | 0.55 |
| ENSG00000214922 | ENSG00000112715 | 0.000 | 0.61 | ENSG00000255737 | ENSG00000168209 | 0.000 | 0.54 |
| ENSG00000214922 | ENSG00000102125 | 0.000 | 0.56 | ENSG00000255737 | ENSG00000117394 | 0.000 | 0.54 |
| ENSG00000215068 | ENSG00000108839 | 0.000 | 0.56 | ENSG00000255737 | ENSG00000105974 | 0.000 | 0.54 |
| ENSG00000215256 | ENSG00000100889 | 0.000 | 0.54 | ENSG00000255737 | ENSG00000059804 | 0.000 | 0.53 |
| ENSG00000215256 | ENSG00000120053 | 0.000 | 0.51 | ENSG00000255737 | ENSG00000166741 | 0.000 | 0.52 |
| ENSG00000215769 | ENSG00000102125 | 0.000 | 0.71 | ENSG00000255737 | ENSG00000147889 | 0.000 | 0.51 |
| ENSG00000215769 | ENSG00000108839 | 0.000 | 0.69 | ENSG00000255746 | ENSG00000176171 | 0.000 | 0.54 |
| ENSG00000215769 | ENSG00000112715 | 0.000 | 0.57 | ENSG00000255746 | ENSG00000147872 | 0.000 | 0.54 |
| ENSG00000222041 | ENSG00000015475 | 0.000 | 0.68 | ENSG00000255746 | ENSG00000100253 | 0.000 | 0.51 |
| ENSG00000222041 | ENSG00000166741 | 0.000 | 0.63 | ENSG00000255774 | ENSG00000147872 | 0.000 | 0.60 |
| ENSG00000222041 | ENSG00000140464 | 0.000 | 0.60 | ENSG00000255774 | ENSG00000176171 | 0.000 | 0.51 |
| ENSG00000222041 | ENSG00000101255 | 0.000 | 0.60 | ENSG00000256128 | ENSG00000015475 | 0.000 | 0.53 |
| ENSG00000222041 | ENSG00000147889 | 0.000 | 0.57 | ENSG00000256128 | ENSG00000147889 | 0.000 | 0.50 |
| ENSG00000222041 | ENSG00000107159 | 0.000 | 0.54 | ENSG00000256540 | ENSG00000115902 | 0.000 | 0.61 |
| ENSG00000222041 | ENSG00000105974 | 0.000 | 0.54 | ENSG00000256540 | ENSG00000112715 | 0.000 | 0.58 |
| ENSG00000222041 | ENSG00000059804 | 0.000 | 0.54 | ENSG00000256546 | ENSG00000101255 | 0.000 | 0.64 |
| ENSG00000222041 | ENSG00000117394 | 0.000 | 0.51 | ENSG00000256546 | ENSG00000015475 | 0.000 | 0.57 |
| ENSG00000222041 | ENSG00000168209 | 0.000 | 0.51 | ENSG00000256546 | ENSG00000099194 | 0.000 | 0.51 |
| ENSG00000222041 | ENSG00000106327 | 0.000 | 0.50 | ENSG00000256546 | ENSG00000168209 | 0.000 | 0.51 |
| ENSG00000223797 | ENSG00000089220 | 0.000 | 0.55 | ENSG00000256546 | ENSG00000171848 | 0.000 | 0.50 |
| ENSG00000223797 | ENSG00000120053 | 0.000 | 0.50 | ENSG00000256546 | ENSG00000147889 | 0.000 | 0.50 |
| ENSG00000224057 | ENSG00000146648 | 0.000 | 0.54 | ENSG00000256694 | ENSG00000115902 | 0.000 | 0.59 |
| ENSG00000224281 | ENSG00000233237 | 0.000 | 0.53 | ENSG00000256694 | ENSG00000112715 | 0.000 | 0.58 |
| ENSG00000224281 | ENSG00000120053 | 0.000 | 0.51 | ENSG00000257556 | ENSG00000107159 | 0.000 | 0.54 |
| ENSG00000224397 | ENSG00000116701 | 0.000 | 0.74 | ENSG00000257556 | ENSG00000102125 | 0.000 | 0.53 |
| ENSG00000224397 | ENSG00000179593 | 0.000 | 0.59 | ENSG00000257702 | ENSG00000100292 | 0.000 | 0.53 |
| ENSG00000224397 | ENSG00000100292 | 0.000 | 0.51 | ENSG00000257989 | ENSG00000112715 | 0.000 | 0.66 |
| ENSG00000224397 | ENSG00000165168 | 0.000 | 0.50 | ENSG00000257989 | ENSG00000108839 | 0.000 | 0.58 |
| ENSG00000224490 | ENSG00000146648 | 0.000 | 0.51 | ENSG00000258548 | ENSG00000146411 | 0.000 | 0.51 |
| ENSG00000224568 | ENSG00000147872 | 0.000 | 0.52 | ENSG00000258667 | ENSG00000112715 | 0.000 | 0.76 |
| ENSG00000224568 | ENSG00000176171 | 0.000 | 0.52 | ENSG00000258667 | ENSG00000135245 | 0.000 | 0.61 |
| ENSG00000224660 | ENSG00000108839 | 0.000 | 0.65 | ENSG00000258667 | ENSG00000108839 | 0.000 | 0.59 |
| ENSG00000224660 | ENSG00000102125 | 0.000 | 0.63 | ENSG00000258727 | ENSG00000102125 | 0.000 | 0.74 |
| ENSG00000224660 | ENSG00000112715 | 0.000 | 0.52 | ENSG00000258727 | ENSG00000108839 | 0.000 | 0.72 |
| ENSG00000224843 | ENSG00000166741 | 0.000 | 0.53 | ENSG00000258727 | ENSG00000112715 | 0.000 | 0.52 |
| ENSG00000224843 | ENSG00000107159 | 0.000 | 0.50 | ENSG00000259172 | ENSG00000146648 | 0.000 | 0.50 |
| ENSG00000224843 | ENSG00000102125 | 0.000 | 0.50 | ENSG00000259291 | ENSG00000120053 | 0.000 | 0.53 |
| ENSG00000224914 | ENSG00000198793 | 0.000 | 0.51 | ENSG00000259291 | ENSG00000089220 | 0.000 | 0.51 |
| ENSG00000225032 | ENSG00000108839 | 0.000 | 0.66 | ENSG00000259347 | ENSG00000147872 | 0.000 | 0.62 |
| ENSG00000225032 | ENSG00000102125 | 0.000 | 0.64 | ENSG00000259347 | ENSG00000176171 | 0.000 | 0.51 |
| ENSG00000225032 | ENSG00000112715 | 0.000 | 0.50 | ENSG00000259347 | ENSG00000100292 | 0.000 | 0.50 |
| ENSG00000225138 | ENSG00000108839 | 0.000 | 0.58 | ENSG00000259834 | ENSG00000165168 | 0.000 | 0.60 |
| ENSG00000225138 | ENSG00000112715 | 0.000 | 0.53 | ENSG00000259834 | ENSG00000118503 | 0.000 | 0.57 |
| ENSG00000225174 | ENSG00000112715 | 0.000 | 0.57 | ENSG00000259834 | ENSG00000116701 | 0.000 | 0.56 |
| ENSG00000225174 | ENSG00000135245 | 0.000 | 0.53 | ENSG00000259865 | ENSG00000102125 | 0.000 | 0.67 |
| ENSG00000225313 | ENSG00000108839 | 0.000 | 0.65 | ENSG00000259865 | ENSG00000108839 | 0.000 | 0.50 |
| ENSG00000225313 | ENSG00000102125 | 0.000 | 0.57 | ENSG00000259969 | ENSG00000146411 | 0.000 | 0.52 |
| ENSG00000225313 | ENSG00000112715 | 0.000 | 0.54 | ENSG00000259969 | ENSG00000155066 | 0.000 | 0.51 |
| ENSG00000225329 | ENSG00000100253 | 0.000 | 0.53 | ENSG00000259972 | ENSG00000102125 | 0.000 | 0.71 |
| ENSG00000225733 | ENSG00000068366 | 0.000 | 0.65 | ENSG00000259972 | ENSG00000108839 | 0.000 | 0.63 |
| ENSG00000225733 | ENSG00000100644 | 0.000 | 0.64 | ENSG00000260121 | ENSG00000108839 | 0.000 | 0.71 |
| ENSG00000225733 | ENSG00000146411 | 0.000 | 0.59 | ENSG00000260121 | ENSG00000112715 | 0.000 | 0.59 |
| ENSG00000225733 | ENSG00000198793 | 0.000 | 0.55 | ENSG00000260121 | ENSG00000102125 | 0.000 | 0.59 |
| ENSG00000225783 | ENSG00000102125 | 0.000 | 0.61 | ENSG00000261051 | ENSG00000101255 | 0.000 | 0.63 |
| ENSG00000225783 | ENSG00000108839 | 0.000 | 0.55 | ENSG00000261051 | ENSG00000117394 | 0.000 | 0.61 |
| ENSG00000225783 | ENSG00000140464 | 0.000 | 0.52 | ENSG00000261051 | ENSG00000135245 | 0.000 | 0.59 |
| ENSG00000225855 | ENSG00000102125 | 0.000 | 0.74 | ENSG00000261051 | ENSG00000166741 | 0.000 | 0.58 |
| ENSG00000225855 | ENSG00000108839 | 0.000 | 0.72 | ENSG00000261051 | ENSG00000059804 | 0.000 | 0.55 |
| ENSG00000225855 | ENSG00000112715 | 0.000 | 0.53 | ENSG00000261051 | ENSG00000099194 | 0.000 | 0.54 |
| ENSG00000225889 | ENSG00000108839 | 0.000 | 0.56 | ENSG00000261051 | ENSG00000015475 | 0.000 | 0.52 |
| ENSG00000225889 | ENSG00000102125 | 0.000 | 0.56 | ENSG00000261051 | ENSG00000105974 | 0.000 | 0.51 |
| ENSG00000226332 | ENSG00000102125 | 0.000 | 0.75 | ENSG00000261051 | ENSG00000168209 | 0.000 | 0.50 |
| ENSG00000226332 | ENSG00000108839 | 0.000 | 0.60 | ENSG00000261087 | ENSG00000102125 | 0.000 | 0.56 |
| ENSG00000226419 | ENSG00000102125 | 0.000 | 0.67 | ENSG00000261170 | ENSG00000147872 | 0.000 | 0.55 |
| ENSG00000226419 | ENSG00000108839 | 0.000 | 0.55 | ENSG00000261170 | ENSG00000176171 | 0.000 | 0.53 |
| ENSG00000226419 | ENSG00000140464 | 0.000 | 0.55 | ENSG00000261175 | ENSG00000176171 | 0.000 | 0.63 |
| ENSG00000226419 | ENSG00000112715 | 0.000 | 0.51 | ENSG00000261175 | ENSG00000147872 | 0.000 | 0.60 |
| ENSG00000226419 | ENSG00000015475 | 0.000 | 0.50 | ENSG00000261175 | ENSG00000100292 | 0.000 | 0.54 |
| ENSG00000226696 | ENSG00000108839 | 0.000 | 0.75 | ENSG00000261175 | ENSG00000099194 | 0.000 | 0.52 |
| ENSG00000226696 | ENSG00000102125 | 0.000 | 0.73 | ENSG00000261175 | ENSG00000135245 | 0.000 | 0.51 |
| ENSG00000226696 | ENSG00000112715 | 0.000 | 0.57 | ENSG00000261183 | ENSG00000089220 | 0.000 | 0.62 |
| ENSG00000226733 | ENSG00000100253 | 0.000 | 0.56 | ENSG00000261183 | ENSG00000120053 | 0.000 | 0.57 |
| ENSG00000226816 | ENSG00000155066 | 0.000 | 0.50 | ENSG00000261324 | ENSG00000102125 | 0.000 | 0.64 |
| ENSG00000227039 | ENSG00000102125 | 0.000 | 0.70 | ENSG00000261324 | ENSG00000108839 | 0.000 | 0.62 |
| ENSG00000227039 | ENSG00000108839 | 0.000 | 0.58 | ENSG00000261326 | ENSG00000102125 | 0.000 | 0.74 |
| ENSG00000227039 | ENSG00000140464 | 0.000 | 0.52 | ENSG00000261326 | ENSG00000108839 | 0.000 | 0.64 |
| ENSG00000227039 | ENSG00000012779 | 0.000 | 0.52 | ENSG00000261326 | ENSG00000112715 | 0.000 | 0.52 |
| ENSG00000227502 | ENSG00000108839 | 0.000 | 0.58 | ENSG00000261490 | ENSG00000102125 | 0.000 | 0.61 |
| ENSG00000227502 | ENSG00000112715 | 0.000 | 0.52 | ENSG00000261490 | ENSG00000108839 | 0.000 | 0.58 |
| ENSG00000227502 | ENSG00000102125 | 0.000 | 0.50 | ENSG00000261505 | ENSG00000108839 | 0.000 | 0.72 |
| ENSG00000227533 | ENSG00000117394 | 0.000 | 0.50 | ENSG00000261505 | ENSG00000102125 | 0.000 | 0.68 |
| ENSG00000228271 | ENSG00000112715 | 0.000 | 0.60 | ENSG00000261505 | ENSG00000112715 | 0.000 | 0.59 |
| ENSG00000228271 | ENSG00000108839 | 0.000 | 0.60 | ENSG00000261634 | ENSG00000233237 | 0.000 | 0.62 |
| ENSG00000228271 | ENSG00000102125 | 0.000 | 0.54 | ENSG00000261634 | ENSG00000120053 | 0.000 | 0.57 |
| ENSG00000228271 | ENSG00000135245 | 0.000 | 0.53 | ENSG00000261634 | ENSG00000089220 | 0.000 | 0.53 |
| ENSG00000228271 | ENSG00000168209 | 0.000 | 0.51 | ENSG00000261888 | ENSG00000155066 | 0.000 | 0.59 |
| ENSG00000228315 | ENSG00000102125 | 0.000 | 0.63 | ENSG00000261971 | ENSG00000102125 | 0.000 | 0.74 |
| ENSG00000228315 | ENSG00000108839 | 0.000 | 0.63 | ENSG00000261971 | ENSG00000108839 | 0.000 | 0.73 |
| ENSG00000228393 | ENSG00000102125 | 0.000 | 0.73 | ENSG00000261971 | ENSG00000112715 | 0.000 | 0.60 |
| ENSG00000228393 | ENSG00000108839 | 0.000 | 0.55 | ENSG00000262089 | ENSG00000108839 | 0.000 | 0.69 |
| ENSG00000228888 | ENSG00000147872 | 0.000 | 0.51 | ENSG00000262089 | ENSG00000102125 | 0.000 | 0.66 |
| ENSG00000229152 | ENSG00000108839 | 0.000 | 0.63 | ENSG00000262089 | ENSG00000107159 | 0.000 | 0.60 |
| ENSG00000229152 | ENSG00000102125 | 0.000 | 0.55 | ENSG00000262089 | ENSG00000112715 | 0.000 | 0.50 |
| ENSG00000229152 | ENSG00000112715 | 0.000 | 0.54 | ENSG00000262580 | ENSG00000102125 | 0.000 | 0.75 |
| ENSG00000229457 | ENSG00000176171 | 0.000 | 0.50 | ENSG00000262580 | ENSG00000108839 | 0.000 | 0.69 |
| ENSG00000230148 | ENSG00000102125 | 0.000 | 0.68 | ENSG00000262877 | ENSG00000102125 | 0.000 | 0.64 |
| ENSG00000230148 | ENSG00000108839 | 0.000 | 0.57 | ENSG00000262877 | ENSG00000108839 | 0.000 | 0.63 |
| ENSG00000230551 | ENSG00000102125 | 0.000 | 0.62 | ENSG00000262877 | ENSG00000112715 | 0.000 | 0.50 |
| ENSG00000230551 | ENSG00000108839 | 0.000 | 0.59 | ENSG00000263004 | ENSG00000089220 | 0.000 | 0.61 |
| ENSG00000230551 | ENSG00000112715 | 0.000 | 0.55 | ENSG00000263004 | ENSG00000120053 | 0.000 | 0.50 |
| ENSG00000231210 | ENSG00000100253 | 0.000 | 0.65 | ENSG00000263272 | ENSG00000102125 | 0.000 | 0.72 |
| ENSG00000231210 | ENSG00000100889 | 0.000 | 0.58 | ENSG00000263272 | ENSG00000108839 | 0.000 | 0.70 |
| ENSG00000231210 | ENSG00000089220 | 0.000 | 0.52 | ENSG00000263272 | ENSG00000112715 | 0.000 | 0.53 |
| ENSG00000231607 | ENSG00000102125 | 0.000 | 0.58 | ENSG00000264112 | ENSG00000102125 | 0.000 | 0.63 |
| ENSG00000231607 | ENSG00000112715 | 0.000 | 0.51 | ENSG00000264112 | ENSG00000108839 | 0.000 | 0.62 |
| ENSG00000231607 | ENSG00000108839 | 0.000 | 0.50 | ENSG00000264456 | ENSG00000108839 | 0.000 | 0.61 |
| ENSG00000231856 | ENSG00000120053 | 0.000 | 0.55 | ENSG00000264456 | ENSG00000102125 | 0.000 | 0.55 |
| ENSG00000231856 | ENSG00000233237 | 0.000 | 0.55 | ENSG00000264920 | ENSG00000089220 | 0.000 | 0.51 |
| ENSG00000231890 | ENSG00000112715 | 0.000 | 0.57 | ENSG00000265519 | ENSG00000198793 | 0.000 | 0.53 |
| ENSG00000231890 | ENSG00000135245 | 0.000 | 0.54 | ENSG00000265519 | ENSG00000068366 | 0.000 | 0.51 |
| ENSG00000231890 | ENSG00000107159 | 0.000 | 0.53 | ENSG00000265743 | ENSG00000140464 | 0.000 | 0.53 |
| ENSG00000231890 | ENSG00000147872 | 0.000 | 0.51 | ENSG00000265743 | ENSG00000012779 | 0.000 | 0.50 |
| ENSG00000232153 | ENSG00000108839 | 0.000 | 0.68 | ENSG00000265962 | ENSG00000107159 | 0.000 | 0.54 |
| ENSG00000232153 | ENSG00000112715 | 0.000 | 0.64 | ENSG00000265962 | ENSG00000101255 | 0.000 | 0.51 |
| ENSG00000232153 | ENSG00000102125 | 0.000 | 0.50 | ENSG00000266968 | ENSG00000155066 | 0.000 | 0.50 |
| ENSG00000232160 | ENSG00000233237 | 0.000 | 0.62 | ENSG00000267121 | ENSG00000102125 | 0.000 | 0.75 |
| ENSG00000232160 | ENSG00000198793 | 0.000 | 0.58 | ENSG00000267121 | ENSG00000108839 | 0.000 | 0.66 |
| ENSG00000232807 | ENSG00000140464 | 0.000 | 0.55 | ENSG00000267244 | ENSG00000108839 | 0.000 | 0.76 |
| ENSG00000232807 | ENSG00000168209 | 0.000 | 0.54 | ENSG00000267244 | ENSG00000102125 | 0.000 | 0.72 |
| ENSG00000232807 | ENSG00000102125 | 0.000 | 0.54 | ENSG00000267244 | ENSG00000112715 | 0.000 | 0.57 |
| ENSG00000232807 | ENSG00000166741 | 0.000 | 0.53 | ENSG00000267519 | ENSG00000102125 | 0.000 | 0.59 |
| ENSG00000232807 | ENSG00000106327 | 0.000 | 0.51 | ENSG00000267519 | ENSG00000112715 | 0.000 | 0.56 |
| ENSG00000232807 | ENSG00000091513 | 0.000 | 0.51 | ENSG00000267519 | ENSG00000081041 | 0.000 | 0.55 |
| ENSG00000232807 | ENSG00000015475 | 0.000 | 0.51 | ENSG00000267519 | ENSG00000108839 | 0.000 | 0.53 |
| ENSG00000232807 | ENSG00000059804 | 0.000 | 0.50 | ENSG00000267892 | ENSG00000108839 | 0.000 | 0.65 |
| ENSG00000232931 | ENSG00000102125 | 0.000 | 0.74 | ENSG00000267892 | ENSG00000112715 | 0.000 | 0.61 |
| ENSG00000232931 | ENSG00000108839 | 0.000 | 0.69 | ENSG00000267892 | ENSG00000102125 | 0.000 | 0.59 |
| ENSG00000232931 | ENSG00000112715 | 0.000 | 0.59 | ENSG00000267892 | ENSG00000107159 | 0.000 | 0.53 |
| ENSG00000232956 | ENSG00000102125 | 0.000 | 0.65 | ENSG00000267892 | ENSG00000147872 | 0.000 | 0.51 |
| ENSG00000232956 | ENSG00000101255 | 0.000 | 0.61 | ENSG00000268713 | ENSG00000102125 | 0.000 | 0.63 |
| ENSG00000232956 | ENSG00000015475 | 0.000 | 0.59 | ENSG00000268713 | ENSG00000015475 | 0.000 | 0.59 |
| ENSG00000232956 | ENSG00000147889 | 0.000 | 0.52 | ENSG00000268713 | ENSG00000147889 | 0.000 | 0.55 |
| ENSG00000233038 | ENSG00000116701 | 0.000 | 0.78 | ENSG00000269352 | ENSG00000102125 | 0.000 | 0.78 |
| ENSG00000233038 | ENSG00000165168 | 0.000 | 0.76 | ENSG00000269352 | ENSG00000108839 | 0.000 | 0.69 |
| ENSG00000233038 | ENSG00000012779 | 0.000 | 0.70 | ENSG00000269352 | ENSG00000112715 | 0.000 | 0.52 |
| ENSG00000233038 | ENSG00000179593 | 0.000 | 0.58 | ENSG00000269486 | ENSG00000112715 | 0.000 | 0.57 |
| ENSG00000233038 | ENSG00000171848 | 0.000 | 0.54 | ENSG00000269486 | ENSG00000108839 | 0.000 | 0.52 |
| ENSG00000233038 | ENSG00000100292 | 0.000 | 0.52 | ENSG00000269867 | ENSG00000102125 | 0.000 | 0.67 |
| ENSG00000233038 | ENSG00000026508 | 0.000 | 0.52 | ENSG00000269867 | ENSG00000108839 | 0.000 | 0.50 |
| ENSG00000233038 | ENSG00000015475 | 0.000 | 0.50 | ENSG00000269958 | ENSG00000102125 | 0.000 | 0.63 |
| ENSG00000233038 | ENSG00000140464 | 0.000 | 0.50 | ENSG00000269958 | ENSG00000112715 | 0.000 | 0.50 |
| ENSG00000233101 | ENSG00000155066 | 0.000 | 0.60 | ENSG00000269958 | ENSG00000108839 | 0.000 | 0.50 |
| ENSG00000233237 | ENSG00000233237 | 0.000 | 1.00 | ENSG00000269985 | ENSG00000176171 | 0.000 | 0.51 |
| ENSG00000233251 | ENSG00000136960 | 0.000 | 0.53 | ENSG00000269985 | ENSG00000147872 | 0.000 | 0.50 |
| ENSG00000233251 | ENSG00000112715 | 0.000 | 0.51 | ENSG00000269985 | ENSG00000166741 | 0.000 | 0.50 |
| ENSG00000233593 | ENSG00000101255 | 0.000 | 0.61 | ENSG00000270012 | ENSG00000102125 | 0.000 | 0.73 |
| ENSG00000233593 | ENSG00000015475 | 0.000 | 0.60 | ENSG00000270012 | ENSG00000108839 | 0.000 | 0.66 |
| ENSG00000233593 | ENSG00000171848 | 0.000 | 0.55 | ENSG00000270055 | ENSG00000102125 | 0.000 | 0.70 |
| ENSG00000233593 | ENSG00000166741 | 0.000 | 0.50 | ENSG00000270055 | ENSG00000108839 | 0.000 | 0.57 |
| ENSG00000233593 | ENSG00000147889 | 0.000 | 0.50 | ENSG00000270547 | ENSG00000136960 | 0.000 | 0.67 |
| ENSG00000233610 | ENSG00000100292 | 0.000 | 0.53 | ENSG00000270547 | ENSG00000115902 | 0.000 | 0.53 |
| ENSG00000233610 | ENSG00000107159 | 0.000 | 0.52 | ENSG00000270547 | ENSG00000112715 | 0.000 | 0.50 |
| ENSG00000233610 | ENSG00000176171 | 0.000 | 0.51 | ENSG00000270607 | ENSG00000015475 | 0.000 | 0.56 |
| ENSG00000233695 | ENSG00000108839 | 0.000 | 0.70 | ENSG00000270607 | ENSG00000166741 | 0.000 | 0.53 |
| ENSG00000233695 | ENSG00000102125 | 0.000 | 0.61 | ENSG00000271270 | ENSG00000112715 | 0.000 | 0.50 |
| ENSG00000233695 | ENSG00000112715 | 0.000 | 0.54 | ENSG00000271533 | ENSG00000108839 | 0.000 | 0.59 |
| ENSG00000233725 | ENSG00000155066 | 0.000 | 0.56 | ENSG00000271533 | ENSG00000102125 | 0.000 | 0.52 |
| ENSG00000233901 | ENSG00000185499 | 0.000 | 0.54 | ENSG00000271533 | ENSG00000112715 | 0.000 | 0.51 |
| ENSG00000234290 | ENSG00000108839 | 0.000 | 0.69 | ENSG00000271895 | ENSG00000102125 | 0.000 | 0.66 |
| ENSG00000234290 | ENSG00000112715 | 0.000 | 0.59 | ENSG00000271895 | ENSG00000108839 | 0.000 | 0.64 |
| ENSG00000234290 | ENSG00000102125 | 0.000 | 0.55 | ENSG00000271895 | ENSG00000112715 | 0.000 | 0.51 |
| ENSG00000234380 | ENSG00000166741 | 0.000 | 0.61 | ENSG00000272505 | ENSG00000108839 | 0.000 | 0.64 |
| ENSG00000234380 | ENSG00000012779 | 0.000 | 0.56 | ENSG00000272505 | ENSG00000112715 | 0.000 | 0.57 |
| ENSG00000234380 | ENSG00000117394 | 0.000 | 0.55 | ENSG00000272505 | ENSG00000102125 | 0.000 | 0.56 |
| ENSG00000234380 | ENSG00000106327 | 0.000 | 0.53 | ENSG00000272512 | ENSG00000112715 | 0.000 | 0.57 |
| ENSG00000234741 | ENSG00000107159 | 0.000 | 0.58 | ENSG00000272512 | ENSG00000108839 | 0.000 | 0.53 |
| ENSG00000234883 | ENSG00000102125 | 0.000 | 0.55 | ENSG00000272512 | ENSG00000102125 | 0.000 | 0.50 |
| ENSG00000234883 | ENSG00000140464 | 0.000 | 0.51 | ENSG00000272711 | ENSG00000117394 | 0.000 | 0.58 |
| ENSG00000234883 | ENSG00000118503 | 0.000 | 0.51 | ENSG00000272711 | ENSG00000166741 | 0.000 | 0.55 |
| ENSG00000234912 | ENSG00000108839 | 0.000 | 0.73 | ENSG00000272711 | ENSG00000101255 | 0.000 | 0.54 |
| ENSG00000234912 | ENSG00000102125 | 0.000 | 0.69 | ENSG00000272711 | ENSG00000012779 | 0.000 | 0.53 |
| ENSG00000234912 | ENSG00000112715 | 0.000 | 0.63 | ENSG00000272711 | ENSG00000015475 | 0.000 | 0.53 |
| ENSG00000235033 | ENSG00000112715 | 0.000 | 0.53 | ENSG00000272711 | ENSG00000107159 | 0.000 | 0.51 |
| ENSG00000235033 | ENSG00000091513 | 0.000 | 0.51 | ENSG00000272752 | ENSG00000102125 | 0.000 | 0.77 |
| ENSG00000235257 | ENSG00000233237 | 0.000 | 0.56 | ENSG00000272752 | ENSG00000108839 | 0.000 | 0.66 |
| ENSG00000235531 | ENSG00000176171 | 0.000 | 0.52 | ENSG00000272821 | ENSG00000102125 | 0.000 | 0.81 |
| ENSG00000235531 | ENSG00000099194 | 0.000 | 0.51 | ENSG00000272821 | ENSG00000108839 | 0.000 | 0.57 |
| ENSG00000235703 | ENSG00000102125 | 0.000 | 0.69 | ENSG00000272821 | ENSG00000140464 | 0.000 | 0.50 |
| ENSG00000235703 | ENSG00000108839 | 0.000 | 0.65 | ENSG00000272870 | ENSG00000112715 | 0.000 | 0.66 |
| ENSG00000235888 | ENSG00000112715 | 0.000 | 0.58 | ENSG00000272870 | ENSG00000107159 | 0.000 | 0.64 |
| ENSG00000236017 | ENSG00000102125 | 0.000 | 0.75 | ENSG00000272870 | ENSG00000135245 | 0.000 | 0.61 |
| ENSG00000236017 | ENSG00000108839 | 0.000 | 0.65 | ENSG00000272870 | ENSG00000176171 | 0.000 | 0.57 |
| ENSG00000237181 | ENSG00000015475 | 0.000 | 0.67 | ENSG00000272870 | ENSG00000108839 | 0.000 | 0.53 |
| ENSG00000237181 | ENSG00000140464 | 0.000 | 0.61 | ENSG00000272870 | ENSG00000168209 | 0.000 | 0.52 |
| ENSG00000237181 | ENSG00000166741 | 0.000 | 0.53 | ENSG00000272933 | ENSG00000198793 | 0.000 | 0.56 |
| ENSG00000237181 | ENSG00000147889 | 0.000 | 0.52 | ENSG00000272933 | ENSG00000146411 | 0.000 | 0.50 |
| ENSG00000237181 | ENSG00000101255 | 0.000 | 0.52 | ENSG00000272990 | ENSG00000015475 | 0.000 | 0.54 |
| ENSG00000237181 | ENSG00000102125 | 0.000 | 0.51 | ENSG00000273001 | ENSG00000112715 | 0.000 | 0.62 |
| ENSG00000237181 | ENSG00000171848 | 0.000 | 0.50 | ENSG00000273001 | ENSG00000107159 | 0.000 | 0.60 |
| ENSG00000237352 | ENSG00000101255 | 0.000 | 0.56 | ENSG00000273032 | ENSG00000112715 | 0.000 | 0.64 |
| ENSG00000237352 | ENSG00000105974 | 0.000 | 0.56 | ENSG00000273032 | ENSG00000108839 | 0.000 | 0.57 |
| ENSG00000237352 | ENSG00000099194 | 0.000 | 0.53 | ENSG00000273032 | ENSG00000102125 | 0.000 | 0.51 |
| ENSG00000237352 | ENSG00000168209 | 0.000 | 0.52 | ENSG00000273142 | ENSG00000102125 | 0.000 | 0.79 |
| ENSG00000237352 | ENSG00000166741 | 0.000 | 0.52 | ENSG00000273142 | ENSG00000108839 | 0.000 | 0.60 |
| ENSG00000237352 | ENSG00000015475 | 0.000 | 0.51 | ENSG00000273151 | ENSG00000108839 | 0.000 | 0.68 |
| ENSG00000237352 | ENSG00000135245 | 0.000 | 0.50 | ENSG00000273151 | ENSG00000102125 | 0.000 | 0.53 |
| ENSG00000237424 | ENSG00000102125 | 0.000 | 0.59 | ENSG00000273179 | ENSG00000102125 | 0.000 | 0.51 |
| ENSG00000237424 | ENSG00000140464 | 0.000 | 0.55 | ENSG00000273373 | ENSG00000102125 | 0.000 | 0.71 |
| ENSG00000237499 | ENSG00000118503 | 0.000 | 0.63 | ENSG00000273373 | ENSG00000108839 | 0.000 | 0.64 |
| ENSG00000237499 | ENSG00000112715 | 0.000 | 0.52 | ENSG00000273599 | ENSG00000108839 | 0.000 | 0.72 |
| ENSG00000237499 | ENSG00000108839 | 0.000 | 0.52 | ENSG00000273599 | ENSG00000112715 | 0.000 | 0.65 |
| ENSG00000237499 | ENSG00000102125 | 0.000 | 0.50 | ENSG00000274020 | ENSG00000102125 | 0.000 | 0.58 |
| ENSG00000237686 | ENSG00000112715 | 0.000 | 0.65 | ENSG00000274173 | ENSG00000155066 | 0.000 | 0.62 |
| ENSG00000237686 | ENSG00000107159 | 0.000 | 0.56 | ENSG00000274272 | ENSG00000102125 | 0.000 | 0.79 |
| ENSG00000237686 | ENSG00000135245 | 0.000 | 0.54 | ENSG00000274272 | ENSG00000108839 | 0.000 | 0.51 |
| ENSG00000237686 | ENSG00000147872 | 0.000 | 0.51 | ENSG00000274605 | ENSG00000089220 | 0.000 | 0.52 |
| ENSG00000238045 | ENSG00000102125 | 0.000 | 0.75 | ENSG00000274605 | ENSG00000120053 | 0.000 | 0.52 |
| ENSG00000238045 | ENSG00000108839 | 0.000 | 0.66 | ENSG00000274605 | ENSG00000166123 | 0.000 | 0.51 |
| ENSG00000238164 | ENSG00000108839 | 0.000 | 0.65 | ENSG00000274605 | ENSG00000128965 | 0.000 | 0.50 |
| ENSG00000238164 | ENSG00000102125 | 0.000 | 0.60 | ENSG00000274925 | ENSG00000102125 | 0.000 | 0.75 |
| ENSG00000240731 | ENSG00000108839 | 0.000 | 0.71 | ENSG00000274925 | ENSG00000108839 | 0.000 | 0.66 |
| ENSG00000240731 | ENSG00000102125 | 0.000 | 0.68 | ENSG00000276649 | ENSG00000102125 | 0.000 | 0.50 |
| ENSG00000240731 | ENSG00000112715 | 0.000 | 0.58 | ENSG00000276980 | ENSG00000166741 | 0.000 | 0.56 |
| ENSG00000241288 | ENSG00000108839 | 0.000 | 0.62 | ENSG00000276980 | ENSG00000012779 | 0.000 | 0.52 |
| ENSG00000241288 | ENSG00000102125 | 0.000 | 0.57 | ENSG00000277011 | ENSG00000155066 | 0.000 | 0.53 |
| ENSG00000241288 | ENSG00000112715 | 0.000 | 0.55 | ENSG00000277268 | ENSG00000155066 | 0.000 | 0.58 |
| ENSG00000241769 | ENSG00000102125 | 0.000 | 0.71 | ENSG00000277268 | ENSG00000125144 | 0.000 | 0.50 |
| ENSG00000241769 | ENSG00000108839 | 0.000 | 0.69 | ENSG00000277476 | ENSG00000102125 | 0.000 | 0.71 |
| ENSG00000241769 | ENSG00000112715 | 0.000 | 0.50 | ENSG00000277476 | ENSG00000108839 | 0.000 | 0.56 |
| ENSG00000242086 | ENSG00000102125 | 0.000 | 0.60 | ENSG00000277559 | ENSG00000147872 | 0.000 | 0.56 |
| ENSG00000242086 | ENSG00000108839 | 0.000 | 0.58 | ENSG00000277559 | ENSG00000112715 | 0.000 | 0.55 |
| ENSG00000244701 | ENSG00000102125 | 0.000 | 0.63 | ENSG00000278058 | ENSG00000102125 | 0.000 | 0.55 |
| ENSG00000244701 | ENSG00000112715 | 0.000 | 0.59 | ENSG00000278058 | ENSG00000108839 | 0.000 | 0.54 |
| ENSG00000244701 | ENSG00000108839 | 0.000 | 0.53 | ENSG00000279159 | ENSG00000108839 | 0.000 | 0.59 |
| ENSG00000244879 | ENSG00000108839 | 0.000 | 0.64 | ENSG00000279159 | ENSG00000102125 | 0.000 | 0.57 |
| ENSG00000244879 | ENSG00000102125 | 0.000 | 0.53 | ENSG00000279738 | ENSG00000102125 | 0.000 | 0.75 |
| ENSG00000244968 | ENSG00000089220 | 0.000 | 0.53 | ENSG00000279738 | ENSG00000108839 | 0.000 | 0.57 |
| ENSG00000244968 | ENSG00000233237 | 0.000 | 0.50 | ENSG00000279833 | ENSG00000108839 | 0.000 | 0.75 |
| ENSG00000245164 | ENSG00000108839 | 0.000 | 0.56 | ENSG00000279833 | ENSG00000112715 | 0.000 | 0.61 |
| ENSG00000245164 | ENSG00000102125 | 0.000 | 0.53 | ENSG00000280206 | ENSG00000015475 | 0.000 | 0.63 |
| ENSG00000245164 | ENSG00000118503 | 0.000 | 0.51 | ENSG00000280206 | ENSG00000102125 | 0.000 | 0.63 |
| ENSG00000245532 | ENSG00000108839 | 0.000 | 0.65 | ENSG00000280206 | ENSG00000147889 | 0.000 | 0.59 |
| ENSG00000245532 | ENSG00000102125 | 0.000 | 0.61 | ENSG00000280206 | ENSG00000140464 | 0.000 | 0.54 |
| ENSG00000245573 | ENSG00000233237 | 0.000 | 0.50 | ENSG00000280206 | ENSG00000101255 | 0.000 | 0.52 |
| ENSG00000245573 | ENSG00000089220 | 0.000 | 0.50 | ENSG00000280206 | ENSG00000107159 | 0.000 | 0.50 |
| ENSG00000245910 | ENSG00000015475 | 0.000 | 0.62 | ENSG00000281005 | ENSG00000108839 | 0.000 | 0.63 |
| ENSG00000245970 | ENSG00000102125 | 0.000 | 0.77 | ENSG00000281005 | ENSG00000102125 | 0.000 | 0.59 |
| ENSG00000246100 | ENSG00000165168 | 0.000 | 0.66 | ENSG00000281005 | ENSG00000112715 | 0.000 | 0.59 |
| ENSG00000246100 | ENSG00000012779 | 0.000 | 0.65 | ENSG00000281026 | ENSG00000102125 | 0.000 | 0.70 |
| ENSG00000246100 | ENSG00000116701 | 0.000 | 0.59 | ENSG00000281026 | ENSG00000108839 | 0.000 | 0.68 |
| ENSG00000246100 | ENSG00000171848 | 0.000 | 0.58 | ENSG00000281026 | ENSG00000112715 | 0.000 | 0.58 |
| ENSG00000246100 | ENSG00000026508 | 0.000 | 0.58 | ENSG00000281103 | ENSG00000116701 | 0.000 | 0.55 |
| ENSG00000246451 | ENSG00000108839 | 0.000 | 0.63 | ENSG00000281103 | ENSG00000118503 | 0.000 | 0.55 |
| ENSG00000246451 | ENSG00000112715 | 0.000 | 0.51 | ENSG00000281103 | ENSG00000140464 | 0.000 | 0.54 |
| ENSG00000246859 | ENSG00000108839 | 0.000 | 0.60 | ENSG00000281103 | ENSG00000165168 | 0.000 | 0.52 |
| ENSG00000246859 | ENSG00000112715 | 0.000 | 0.59 | ENSG00000281103 | ENSG00000171848 | 0.000 | 0.51 |
| ENSG00000247095 | ENSG00000107159 | 0.000 | 0.67 | ENSG00000281103 | ENSG00000012779 | 0.000 | 0.51 |
| ENSG00000247095 | ENSG00000112715 | 0.000 | 0.66 | ENSG00000281769 | ENSG00000155066 | 0.000 | 0.63 |

**Table 2 Verified overall survival correlated FR-DELs by univariate Cox analysis**

| **Term** | **P** | **LogRank** | **HR** | **HRlower** | **HRupper** |
| --- | --- | --- | --- | --- | --- |
| ENSG00000179406 | 0.001 | 0.001 | 2.17 | 1.35 | 3.47 |
| ENSG00000180769 | 0.004 | 0.004 | 0.51 | 0.32 | 0.81 |
| ENSG00000183784 | 0.006 | 0.005 | 0.52 | 0.33 | 0.83 |
| ENSG00000187951 | 0.002 | 0.002 | 2.06 | 1.30 | 3.27 |
| ENSG00000196295 | 0.008 | 0.007 | 1.88 | 1.18 | 2.99 |
| ENSG00000196756 | 0.000 | 0.000 | 2.80 | 1.73 | 4.53 |
| ENSG00000197989 | 0.043 | 0.041 | 1.59 | 1.01 | 2.51 |
| ENSG00000206195 | 0.000 | 0.000 | 2.81 | 1.72 | 4.61 |
| ENSG00000215256 | 0.003 | 0.002 | 0.49 | 0.31 | 0.78 |
| ENSG00000215769 | 0.004 | 0.004 | 1.96 | 1.23 | 3.10 |
| ENSG00000224660 | 0.030 | 0.029 | 1.66 | 1.05 | 2.62 |
| ENSG00000225032 | 0.011 | 0.010 | 1.81 | 1.14 | 2.86 |
| ENSG00000225313 | 0.023 | 0.022 | 1.70 | 1.07 | 2.69 |
| ENSG00000225329 | 0.003 | 0.003 | 0.50 | 0.31 | 0.79 |
| ENSG00000225783 | 0.001 | 0.000 | 2.29 | 1.43 | 3.68 |
| ENSG00000225855 | 0.001 | 0.000 | 2.27 | 1.42 | 3.63 |
| ENSG00000226419 | 0.001 | 0.000 | 2.32 | 1.44 | 3.75 |
| ENSG00000226696 | 0.001 | 0.001 | 2.21 | 1.38 | 3.54 |
| ENSG00000226733 | 0.002 | 0.002 | 0.48 | 0.30 | 0.76 |
| ENSG00000228393 | 0.003 | 0.003 | 2.03 | 1.27 | 3.25 |
| ENSG00000229152 | 0.006 | 0.005 | 1.92 | 1.21 | 3.06 |
| ENSG00000230551 | 0.001 | 0.001 | 2.15 | 1.34 | 3.45 |
| ENSG00000231607 | 0.000 | 0.000 | 2.72 | 1.67 | 4.43 |
| ENSG00000231856 | 0.002 | 0.001 | 0.47 | 0.29 | 0.75 |
| ENSG00000232931 | 0.007 | 0.006 | 1.88 | 1.19 | 2.98 |
| ENSG00000233251 | 0.001 | 0.001 | 0.46 | 0.28 | 0.73 |
| ENSG00000233593 | 0.000 | 0.000 | 3.44 | 2.07 | 5.72 |
| ENSG00000234380 | 0.007 | 0.006 | 1.90 | 1.20 | 3.03 |
| ENSG00000234883 | 0.001 | 0.001 | 2.26 | 1.40 | 3.64 |
| ENSG00000235703 | 0.009 | 0.008 | 1.85 | 1.17 | 2.95 |
| ENSG00000236017 | 0.008 | 0.007 | 1.87 | 1.18 | 2.97 |
| ENSG00000237181 | 0.001 | 0.001 | 2.15 | 1.34 | 3.43 |
| ENSG00000237424 | 0.005 | 0.005 | 1.93 | 1.22 | 3.07 |
| ENSG00000241769 | 0.021 | 0.020 | 1.72 | 1.08 | 2.71 |
| ENSG00000245970 | 0.001 | 0.001 | 2.15 | 1.35 | 3.42 |
| ENSG00000246100 | 0.006 | 0.005 | 1.91 | 1.20 | 3.02 |
| ENSG00000247373 | 0.019 | 0.018 | 1.74 | 1.09 | 2.75 |
| ENSG00000247774 | 0.017 | 0.016 | 1.76 | 1.11 | 2.79 |
| ENSG00000247982 | 0.018 | 0.016 | 1.74 | 1.10 | 2.75 |
| ENSG00000248019 | 0.002 | 0.001 | 2.11 | 1.32 | 3.37 |
| ENSG00000248323 | 0.000 | 0.000 | 3.07 | 1.87 | 5.04 |
| ENSG00000248636 | 0.002 | 0.002 | 0.48 | 0.30 | 0.77 |
| ENSG00000248866 | 0.005 | 0.005 | 0.52 | 0.33 | 0.83 |
| ENSG00000249087 | 0.013 | 0.012 | 1.79 | 1.13 | 2.82 |
| ENSG00000249550 | 0.000 | 0.000 | 2.78 | 1.71 | 4.54 |
| ENSG00000249776 | 0.000 | 0.000 | 0.37 | 0.23 | 0.60 |
| ENSG00000249859 | 0.002 | 0.002 | 2.10 | 1.31 | 3.37 |
| ENSG00000251136 | 0.000 | 0.000 | 2.53 | 1.57 | 4.07 |
| ENSG00000251169 | 0.002 | 0.001 | 0.47 | 0.29 | 0.75 |
| ENSG00000254815 | 0.021 | 0.020 | 1.72 | 1.08 | 2.72 |
| ENSG00000255717 | 0.044 | 0.042 | 1.59 | 1.01 | 2.50 |
| ENSG00000255737 | 0.001 | 0.000 | 2.27 | 1.42 | 3.63 |
| ENSG00000255774 | 0.000 | 0.000 | 0.43 | 0.27 | 0.69 |
| ENSG00000256128 | 0.006 | 0.005 | 1.95 | 1.22 | 3.12 |
| ENSG00000256546 | 0.003 | 0.003 | 2.01 | 1.26 | 3.19 |
| ENSG00000259865 | 0.002 | 0.002 | 2.09 | 1.31 | 3.34 |
| ENSG00000259972 | 0.024 | 0.022 | 1.70 | 1.07 | 2.68 |
| ENSG00000261051 | 0.001 | 0.001 | 2.16 | 1.35 | 3.45 |
| ENSG00000261087 | 0.009 | 0.008 | 1.86 | 1.17 | 2.96 |
| ENSG00000261170 | 0.002 | 0.001 | 0.47 | 0.30 | 0.76 |
| ENSG00000261175 | 0.002 | 0.001 | 0.47 | 0.30 | 0.75 |
| ENSG00000261324 | 0.020 | 0.019 | 1.72 | 1.09 | 2.71 |
| ENSG00000261326 | 0.013 | 0.012 | 1.79 | 1.13 | 2.84 |
| ENSG00000261505 | 0.007 | 0.006 | 1.90 | 1.19 | 3.01 |
| ENSG00000261634 | 0.001 | 0.000 | 0.43 | 0.26 | 0.69 |
| ENSG00000262580 | 0.004 | 0.003 | 1.98 | 1.24 | 3.15 |
| ENSG00000262877 | 0.018 | 0.017 | 1.74 | 1.10 | 2.75 |
| ENSG00000263272 | 0.002 | 0.002 | 2.11 | 1.32 | 3.37 |
| ENSG00000264112 | 0.001 | 0.001 | 2.24 | 1.40 | 3.60 |
| ENSG00000264456 | 0.001 | 0.001 | 2.20 | 1.38 | 3.51 |
| ENSG00000267121 | 0.001 | 0.001 | 2.16 | 1.35 | 3.46 |
| ENSG00000267244 | 0.003 | 0.003 | 2.01 | 1.26 | 3.20 |
| ENSG00000268713 | 0.002 | 0.001 | 2.14 | 1.33 | 3.45 |
| ENSG00000269352 | 0.008 | 0.007 | 1.87 | 1.18 | 2.96 |
| ENSG00000269867 | 0.002 | 0.001 | 2.11 | 1.32 | 3.37 |
| ENSG00000270012 | 0.025 | 0.024 | 1.69 | 1.07 | 2.67 |
| ENSG00000270055 | 0.002 | 0.002 | 2.08 | 1.31 | 3.30 |
| ENSG00000271895 | 0.018 | 0.016 | 1.74 | 1.10 | 2.74 |
| ENSG00000272821 | 0.000 | 0.000 | 2.31 | 1.44 | 3.71 |
| ENSG00000272990 | 0.048 | 0.046 | 1.59 | 1.00 | 2.52 |
| ENSG00000273142 | 0.002 | 0.001 | 2.12 | 1.32 | 3.40 |
| ENSG00000273179 | 0.002 | 0.002 | 2.05 | 1.30 | 3.25 |
| ENSG00000273373 | 0.002 | 0.002 | 2.09 | 1.31 | 3.33 |
| ENSG00000274020 | 0.002 | 0.002 | 2.07 | 1.30 | 3.30 |
| ENSG00000274272 | 0.040 | 0.038 | 1.61 | 1.02 | 2.52 |
| ENSG00000274925 | 0.009 | 0.008 | 1.85 | 1.16 | 2.94 |
| ENSG00000279738 | 0.001 | 0.001 | 2.21 | 1.38 | 3.54 |
| ENSG00000280206 | 0.000 | 0.000 | 2.35 | 1.47 | 3.77 |
| ENSG00000281026 | 0.006 | 0.006 | 1.91 | 1.20 | 3.05 |


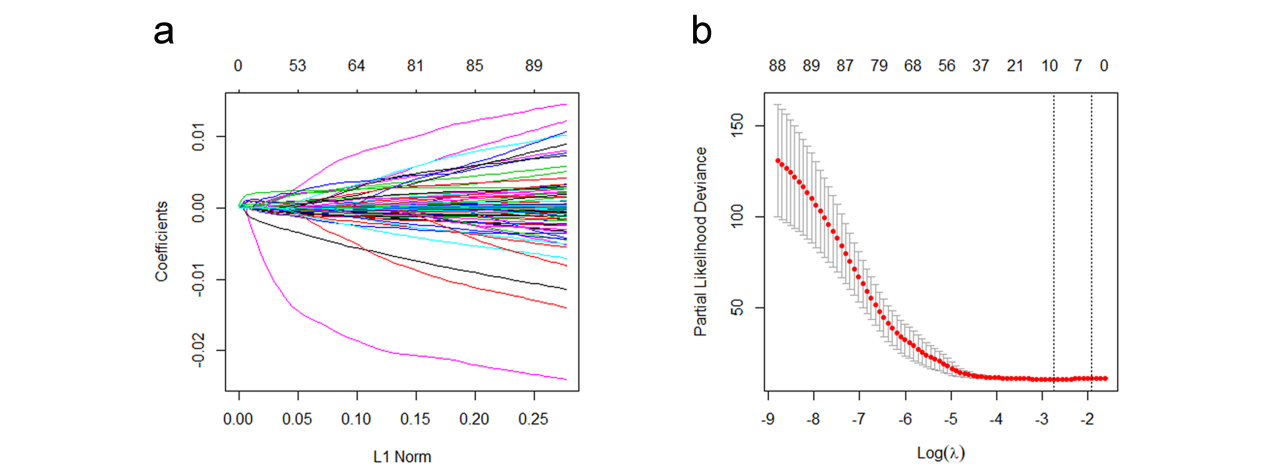


**Supplementary figure 1** LASSO regression analysis for those 89 FR-DELs verified by univariate Cox analyses and Kaplan-Meier analysis.


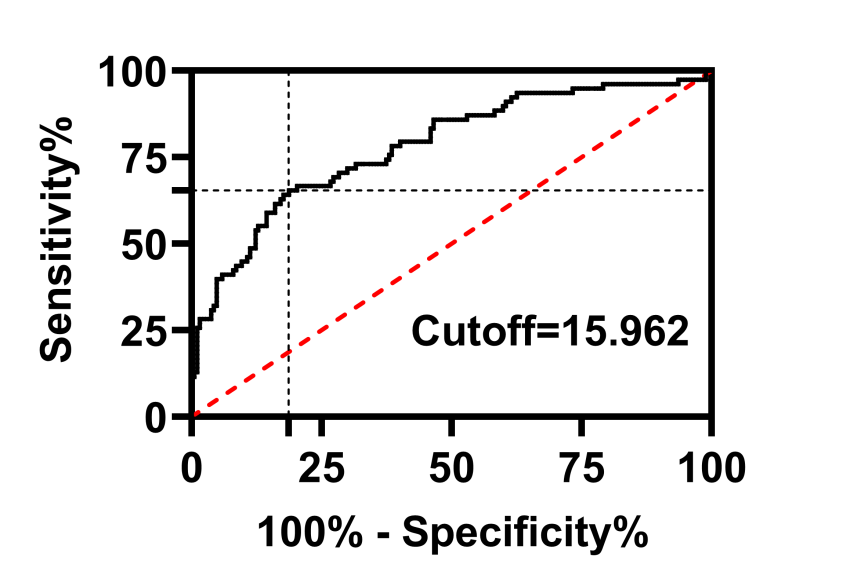


**Supplementary figure 2** The verified optimal cutoff value was 15.962.


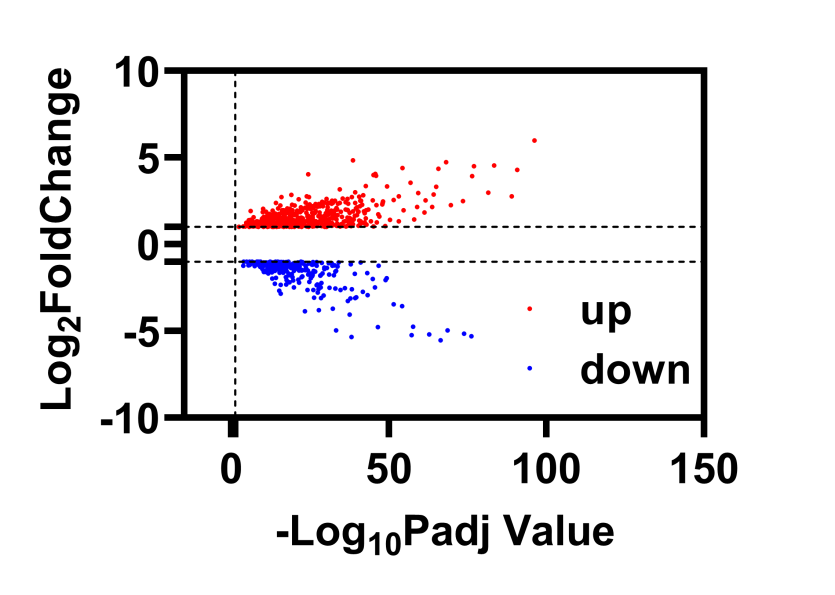


**Supplementary figure 3** Volcano plot of DEGs for KIRC between high risk group and low risk group.
